# A Bacterial Inflammation Sensor Regulates c-di-GMP Signaling, Adhesion, and Biofilm Formation

**DOI:** 10.1128/mBio.00173-21

**Published:** 2021-06-22

**Authors:** Arden Perkins, Dan A. Tudorica, Raphael D. Teixeira, Tilman Schirmer, Lindsay Zumwalt, O. Maduka Ogba, C. Keith Cassidy, Phillip J. Stansfeld, Karen Guillemin

**Affiliations:** a Institute of Molecular Biology, University of Oregon, Eugene, Oregon, USA; b Biozentrum, University of Basel, Basel, Switzerland; c Department of Chemistry and Biochemistry Program, Schmid College of Science and Technology, Chapman University, Orange, California, USA; d Department of Biochemistry, University of Oxford, Oxford, United Kingdom; e School of Life Sciences & Department of Chemistry, University of Warwick, Coventry, United Kingdom; f Humans and the Microbiome Program, CIFAR, Toronto, Ontario, Canada; University of Pittsburgh

**Keywords:** hypochlorous acid, redox signaling, bacterial sensory transduction, chemoreceptor zinc-binding domains, diguanylate cyclase Z, CZB, HOCl, biofilm, bleach, c-di-GMP, diguanylate cyclase, reactive oxygen species

## Abstract

Bacteria that colonize animals must overcome, or coexist, with the reactive oxygen species products of inflammation, a front-line defense of innate immunity. Among these is the neutrophilic oxidant bleach, hypochlorous acid (HOCl), a potent antimicrobial that plays a primary role in killing bacteria through nonspecific oxidation of proteins, lipids, and DNA. Here, we report that in response to increasing HOCl levels, Escherichia coli regulates biofilm production via activation of the diguanylate cyclase DgcZ. We identify the mechanism of DgcZ sensing of HOCl to be direct oxidation of its regulatory chemoreceptor zinc-binding (CZB) domain. Dissection of CZB signal transduction reveals that oxidation of the conserved zinc-binding cysteine controls CZB Zn^2+^ occupancy, which in turn regulates the catalysis of c-di-GMP by the associated GGDEF domain. We find DgcZ-dependent biofilm formation and HOCl sensing to be regulated *in vivo* by the conserved zinc-coordinating cysteine. Additionally, point mutants that mimic oxidized CZB states increase total biofilm. A survey of bacterial genomes reveals that many pathogenic bacteria that manipulate host inflammation as part of their colonization strategy possess CZB-regulated diguanylate cyclases and chemoreceptors. Our findings suggest that CZB domains are zinc-sensitive regulators that allow host-associated bacteria to perceive host inflammation through reactivity with HOCl.

## INTRODUCTION

Host-associated bacteria use a repertoire of sensory proteins to eavesdrop on host chemical cues and adapt their lifestyles accordingly. Two primary pathways that bacteria use for lifestyle adaptation in hosts are biofilm formation (1), regulated by diguanylate cyclases (2), and chemotaxis (3), regulated by chemoreceptors (4), which serve to direct bacterial localization and biogeography. One of the most consequential host processes for bacteria that colonize animals is inflammation, in which neutrophils infiltrate tissue and generate reactive oxygen and nitrogen species to control and eliminate invading microbes (5, 6). Of these cytotoxic species, hypochlorous acid (HOCl) has been shown to play a dominant role in killing bacteria (7). HOCl is catalyzed from hydrogen peroxide (H_2_O_2_) and chloride by myeloperoxidase, an enzyme abundant in neutrophil granules and neutrophil extracellular traps (8, 9). Inflamed tissue can harbor millimolar concentrations of HOCl, with individual neutrophils able to generate as much as 134 mM HOCl/min (6, 10–12). HOCl is an extremely reactive chemical that acts as a bactericide through the oxidation of a broad spectrum of cellular components, especially sulfur-containing amino acids (5, 7, 13). A cellular consequence of HOCl exposure is the mobilization of cellular zinc, which occurs as a result of HOCl oxidation of zinc-cysteine clusters and small-molecule thiols (14–16).

Prior investigations have sought to understand bacterial defenses against HOCl, which include upregulation of antioxidant enzymes through the HOCl-responsive transcription factors OxyR, HypT, RclR, and NemR (17–21) and activation of the molecular chaperones Hsp33 and CnoX (22, 23). However, the idea that bacteria could utilize HOCl as a chemical cue to inform decisions about sessility, motility, and localization within a host is comparatively understudied. Yet, many examples are known in which bacteria utilize biofilm and chemotaxis pathways to colonize inflamed tissue. For example, the formation of biofilms inhibits bacterial clearance even in the face of extensive neutrophil recruitment, such as for uropathogenic Escherichia coli strains that cause pyuria (24), and *Legionella* spp. and Streptococcus pneumoniae strains that establish persistent infections in the inflamed lung (25–27). The enteric pathogen Salmonella enterica serovar Typhimurium utilizes chemoattraction toward the alternative electron acceptor tetrathionate, formed by reaction of HOCl and hydrogen sulfide in the inflamed gut, to outcompete native obligate fermenters of the microbiota (28). The human stomach pathogen Helicobacter pylori uses chemotaxis to localize to gastric wounds (29) and colonizes the stomach antrum and corpus despite an aggressive inflammation response (30). In addition, individuals with chronic gastrointestinal inflammation, such as Crohn’s disease and ulcerative colitis, are well known to exhibit disrupted microbiome communities thought to perpetuate a cycle of dysbiosis recalcitrant to correction with therapeutics (31, 32). These examples suggest that inflamed host environments that contain HOCl can dramatically shift the colonization, biogeography, and behavior of host-associated bacteria.

Chemoreceptor zinc-binding (CZB) protein domains were first identified in H. pylori (33) and consist of a four-helix bundle fold with a unique and conserved 3His/1Cys zinc-binding motif (34). CZB-containing proteins have been reported to play roles in sensing a diverse set of exogenous effectors, including Zn^2+^ and other metals, pH, nutrients, H_2_O_2_, and superoxide, to regulate bacterial chemoreceptors and diguanylate cyclases (33–40). Whether CZB domains use a shared molecular mechanism to mediate cellular responses to such dissimilar stimuli has remained unclear. Adding to the enigma of CZB ligand sensing, we recently showed that H. pylori uses the chemoreceptor transducer-like protein D (TlpD), which contains a C-terminal CZB domain, to sense HOCl through direct oxidation of the conserved Cys residue in the CZB zinc-binding core (35). As an example of the biological significance of this mechanism and relevance to human disease, TlpD was shown to facilitate chemoattraction to HOCl sources for H. pylori, providing an explanation for the bacterium’s persistence in inflamed tissue and tropism for gastric wounds (29, 30, 35, 41).

H. pylori is an unusual bacterium that is highly adapted for colonizing the human stomach, and it is possible that our observation of HOCl sensing by TlpD was a function unique to a single H. pylori chemoreceptor. Thus, it was unclear to what extent our findings on the chemoreceptor TlpD could be extrapolated to the diverse array of CZB-containing proteins that exist in nature, such as CZB-regulated diguanylate cyclases which, unlike TlpD, contain an N-terminally-linked CZB (34). Distant CZB homologues exhibit important differences, including low sequence similarity outside the zinc-binding core and cytoplasmic versus periplasmic cellular localization. Additionally, CZB-containing proteins are possessed by bacteria that inhabit diverse environments (33–35). In fact, prior evidence suggested that CZB domains in other contexts might function solely as direct sensors of exogenous Zn^2+^. The Escherichia coli diguanylate cyclase Z (DgcZ, previously referred to as YdeH) (34, 42–44) is a cytosolic CZB-regulated diguanylate cyclase and catalyzes the conversion of GTP to bis-(3′–5′)-cyclic dimeric GMP (c-di-GMP), a ubiquitous bacterial signaling molecule well known to play pivotal roles in bacterial decisions of cell adhesion, biofilm formation, and pathogenicity (1, 45, 46). Generally, increases in cellular c-di-GMP correspond to decreased motility, synthesis of biofilm polymers, and formation of biofilm (45). Earlier work showed that E. coli DgcZ catalysis of c-di-GMP is regulated through a subfemtomolar affinity for Zn^2+^, whereby the zinc-bound protein is enzymatically inhibited and the zinc-free protein is activated, and addition of exogenous Zn^2+^ reduces the formation of biofilm (34). E. coli DgcZ exhibits many differences from H. pylori TlpD: the CZB domains of the two proteins share only 20% sequence similarity, the full-length proteins have different domain architectures and signaling outputs, and the bacteria colonize different sites within the host. Additionally, E. coli does not possess a TlpD-like chemoreceptor. Thus, we have used E. coli DgcZ as a model system to investigate whether CZB domains act as HOCl sensors in diverse contexts to regulate host-associated bacterial lifestyles.

In this study, we contribute to an emerging picture of how bacterial CZB domains act as monitors of cellular zinc homeostasis and how host-associated bacteria can utilize this function to perceive the neutrophilic oxidant HOCl, a major disruptor of zinc homeostasis (15, 16). These data support earlier reports that oxidants can increase E. coli biofilm formation (38, 47) and suggest how enteropathogenic, enteroaggregative, and uropathogenic E. coli may respond to host inflammation to favor pathogenicity (48–50). The ability of CZB domains to sense HOCl and regulate host-associated bacterial lifestyles implicates this family of proteins as important players in diseases of gut dysbiosis and chronic inflammation, with relevance for bacterial biology across diverse phyla.

## RESULTS

### DgcZ regulates biofilm in response to exogenous HOCl.

**FIG 1:**
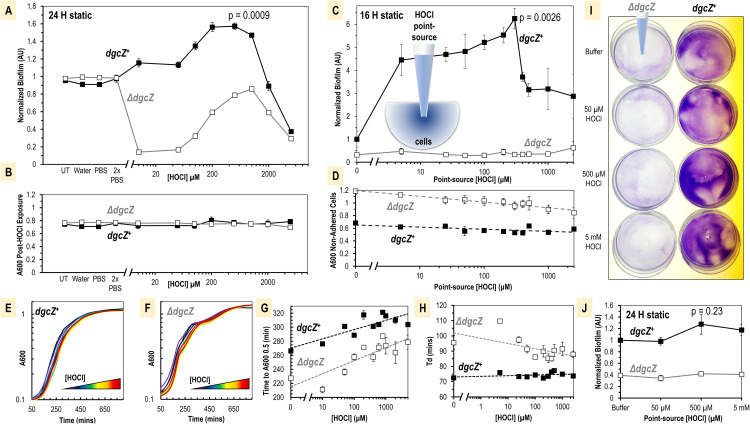
DgcZ regulation of biofilm formation in response to HOCl. (A) Biofilm after 24 h of static growth at 30°C following exposure to controls or HOCl treatments diluted in PBS (*n* = 4). Cell cultures were grown in a 96-well microplate with an initial *A*_600_ of 0.5 at the time of treatment. (B) Final *A*_600_ of cultures described for panel A. (C) Biofilm after 16 h at 25°C with exposure to pipettes containing various concentrations of HOCl point sources (*n* = 4). (D) Final *A*_600_ of planktonic fraction of cultures described for panel C. (E and F) Average growth curves are shown following treatments described for panels C and D (*n* = 4). A 180-μl volume of LB was inoculated with 20 ml of cultures and grown at 37°C. Lines are colored as follows: buffer, black; 5 μM HOCl, purple; 25 μM HOCl, indigo; 50 μM HO, dark blue; 100 μM HOCl, blue; 200 μM HOCl, dark green; 300 μM HOCl, light green; 400 μM HOCl, yellow; 500 μM HOCl, orange; 1,000 μM HOCl, red; 2,500 μM HOCl, dark red. (G) Quantification of the time required for cultures described for panels E and F to reach an *A*_600_ of 0.5. (H) Doubling time (Td) during exponential growth of cultures described for panels E and F. (I) Representative results, from four replicates, for 24-h static biofilm assays with 3 ml of cell culture grown at 25°C with a central point source treatment following staining of adhered cells with crystal violet. (J) Quantification of biofilm described for panel I (*n* = 4). For all data shown, points indicate the sample means, and error bars are standard errors of the mean. All data values are available in Data Set S1A in the supplemental material. *P* values from paired Student *t* tests for *dgcZ^+^* untreated and peak biofilm responses are indicated.

To investigate whether E. coli DgcZ could function as an HOCl sensor and regulate bacterial biofilm in response to HOCl, E. coli biofilms were grown and quantified under various conditions. When grown *in vitro*, E. coli DgcZ expression is inhibited by carbon storage regulator A (CsrA), which directly binds mRNA transcripts of DgcZ to inhibit translation (43, 51). Therefore, we utilized a previously engineered *csrA* deletion strain of MG1655 that permits a moderate degree of DgcZ expression and biofilm formation under laboratory conditions to mimic behavior inside a host (43). In this background, we compared the biofilm formation of strains expressing or lacking *dgcZ* (*dgcZ^+^* or Δ*dgcZ*, respectively) in a static microplate when treated with increasing concentrations of HOCl at mid-log exponential phase (Fig. 1A). Crystal violet staining was used to quantify biofilm as done previously (34), with relative biofilm calculated as a ratio of the biofilm of each sample divided by the average biofilm of untreated wild type in the same experiment. No difference in biofilm was observed after 24 h for controls containing untreated cells, for water-treated strains, or for strains treated with phosphate-buffered saline (PBS) buffer at pH 7 (Fig. 1A). Single applications of HOCl in PBS buffer showed a bimodal response for wild-type cells, with relative biofilm increasing in response to micromolar HOCl, to a maximum of 1.6-fold at 250 μM, and decreasing at higher HOCl concentrations (Fig. 1A). The Δ*dgcZ* mutant displayed a different trend, showing decreased biofilm in the range of 5 to 250 μM HOCl (Fig. 1A). The significance of non-*dgcZ*-dependent biofilm changes was unclear and not replicated in other experiment formats (see below), so we did not investigate it further. Under these conditions, both *dgcZ^+^* and Δ*dgcZ* cultures were observed to grow equivalently and did not display growth inhibition (Fig. 1B). Experiments with more established cultures with cells treated at an *A*_600_ of 1.0 showed a smaller *dgcZ*-dependent increase in biofilm in response to a single HOCl treatment, as well as biofilm inhibition with exogenous zinc treatment, as was previously reported (34) (see Fig. S1A in the supplemental material).

10.1128/mBio.00173-21.1 FIG S1 E. coli biofilm formation in response to HOCl. (A) Biofilm formation with treatments performed at an *A*_600_ of 1.0. Cells were dispensed in a 96-well microplate, treated as indicated, and grown statically for 24 h in LB at 30°C (*n* = 4). Data shown are sample means, and error bars are standard errors of the mean. A *P* value from a paired Student *t* test is shown between 100 μM HOCl treatment and buffer treatment control. (B) Previous mechanism of CZB HOCl sensing proposed by Perkins et al. (35). Note that we refine this model later in the study (Fig. 7). Download FIG S1, TIF file, 1.3 MB. Copyright © 2021 Perkins et al. 2021 Perkins et al. https://creativecommons.org/licenses/by/4.0/ This content is distributed under the terms of the Creative Commons Attribution 4.0 International license.

10.1128/mBio.00173-21.10 DATA SET S1 Data from this work. (A) Data values for main text and supplemental figures. (B) Data and information relating to quantum mechanics calculations. (C) Database of CZB-containing proteins. See attached spreadsheet. Download Data Set S1, XLSX file, 4.5 MB. Copyright © 2021 Perkins et al. 2021 Perkins et al. https://creativecommons.org/licenses/by/4.0/ This content is distributed under the terms of the Creative Commons Attribution 4.0 International license.

Since single treatments of HOCl might react quickly and dissipate, we performed a similar version of this static biofilm microplate assay with treatment “point sources” to model a more sustained exposure to an HOCl microgradient, such as might occur in the vicinity of neutrophils and extracellular granules that contain myeloperoxidase. This was accomplished by using a 96-well Rainin liquidator with pipettes containing HOCl treatments over the range of 5 to 2,500 μM that were submerged in cell cultures for 16 h (Fig. 1C). These data showed a *dgcZ*-dependent maximal increase in relative biofilm of 6.25-fold at 300 μM (Fig. 1C). For these cultures, the *A*_600_ of the planktonic population was lower for the *dgcZ^+^* than the Δ*dgcZ* strain, possibly owing to the higher fraction of surface-attached cells for the *dgcZ^+^* strain, and a slight negative correlation was observed between cell density and increasing HOCl concentrations (Fig. 1D). To test the growth of cells post-HOCl treatment, we inoculated fresh LB with cultures from HOCl point source experiment endpoints (Fig. 1E and F). These experiments showed a slight delay in time to reach mid-log exponential phase as a function of increasing HOCl concentrations (Fig. 1G), but the doubling times during exponential phase were similar across a large range of HOCl treatments (Fig. 1H). The influence on biofilm as a function of proximity to an HOCl microgradient was difficult to assess using microliter volumes of cell culture, so we scaled up these experiments in 15-mm petri dishes and 3 ml of cell culture. Experiments contained a central point source treatment of buffer or HOCl, and biofilms were visualized by crystal violet staining after 24 h. These assays suggested that biofilm formation distribution may be altered in response to HOCl microgradients (Fig. 1I). The total biofilm tended to increase in response to HOCl in these assays, similar to experiments with smaller cell volumes (Fig. 1J). Collectively, these data indicate that DgcZ regulates biofilm formation in response to physiological concentrations of HOCl and that no serious toxicity or impediment to growth occurs for E. coli under these conditions.

### DgcZ uses a zinc-binding cysteine for selective reactivity with HOCl.

**FIG 2:**
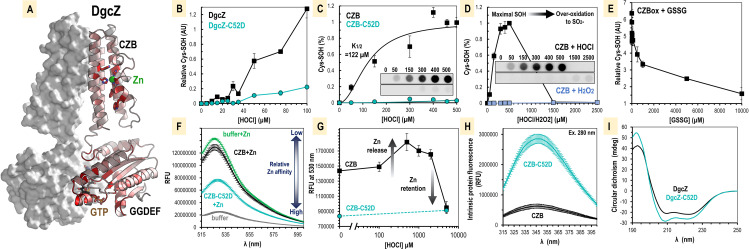
Oxidation of E. coli DgcZ by HOCl and influence on protein zinc ligation. (A) The DgcZ homodimer is shown, with each monomer containing a C-terminal GGDEF domain to catalyze c-di-GMP from GTP and an N-terminal CZB domain that allosterically regulates the GGDEF domain through zinc-mediated inhibition (34). To highlight regions of functional importance, amino acid conservation across DgcZ-like homologues containing similar architecture is depicted, colored by conservation from 0 to 100% (white to red). (B) C52 is required for efficient reactivity with HOCl for full-length DgcZ (wild type shown in black, C52D mutant shown in teal; *n* = 3). Reactions were performed in PBS at pH 7 with 10 μM protein. Data points are the averages across replicate samples, and error bars are standard errors of the mean. (C) The CZB domain alone is sufficient to recapitulate C52-mediated HOCl reactivity. Representative Western blots are shown for 10 μM wild-type CZB (top) and CZB-C52D (bottom) (*n* = 8). A fit of the Hill equation to the wild-type (black line) and C52D mutant (teal line) data with a Hill coefficient of 2 is shown and used to calculate the concentration of HOCl required for half-maximal Cys-SOH formation (*K*_1/2_). (D) The CZB domain (10 μM) is preferentially reactive with HOCl over H_2_O_2_, and the C52 mutant is likely overoxidized to form Cys-SO_2_^−^ at high concentrations (*n* = 3). (E) Oxidized CZB protein (10 μM) pretreated with 250 μM HOCl can be reduced by glutathione disulfide (*n* = 7 or 8). (F) Emission spectra showing competition for zinc between zinpyr-1 probe and CZB proteins (*n* = 3). Reaction mixtures contained 10 μM probe and 1 μM protein with or without 5 μM ZnSO_4_ in PBS buffer, pH 7. Zinpyr-1 emission spectra were obtained by excitation at 488 nm. (G) Emission spectra showing Zn^2+^ release or retention is regulated by CZB oxidation state in the presence of zinpyr-1. Reaction mixtures contained 10 μM protein in PBS, pH 7 (*n* = 3). Samples were treated with the indicated concentrations of HOCl and then quenched with 1 mM methionine, followed by addition of 10 μM probe. Spectra shown are blank subtracted from equivalent treatments without protein addition. (H) Intrinsic fluorescence spectra of 10 μM wild-type CZB and CZB-C52D in PBS (*n* = 3), with excitation at 280 nm. (I) A circular dichroism spectra for 5 μM DgcZ wild type and DgcZ-C52D in PBS. All data values are available in Data Set S1A in the supplemental material. AU, arbitrary units; mdeg, millidegrees; RFU, relative fluorescence units.

Previously, we demonstrated that the H. pylori chemoreceptor TlpD detects the inflammation product HOCl through direct oxidation of the cysteine of the zinc-binding core to form cysteine sulfenic acid (Cys-SOH) (Fig. S1B) (35, 52). The structure of E. coli DgcZ is comprised of a CZB domain (residues 1 to 128) and a GGDEF domain (residues 129 to 296) (Fig. 2A). The CZB domain of DgcZ contains a 3His/1Cys zinc-binding core identical to that of TlpD (34, 35, 52) and so could, in principle, sense HOCl by the same molecular mechanism. Mapping amino acid conservation patterns of CZB-regulated diguanylate cyclases (232 sequences) onto the crystal structure of E. coli DgcZ (models based on PDB ID 3T9O and 4H54) (34) showed strong conservation in the regions proximal to the CZB zinc-binding site and the GGDEF catalytic site, suggesting that these regions of the protein are broadly important for function (Fig. 2A).

To investigate the molecular basis for DgcZ sensing of exogenous HOCl and regulation of biofilm, we conducted biochemical analyses with purified recombinant E. coli DgcZ to experimentally determine how the protein could facilitate HOCl sensing. We used DgcZ mutants as tools to model the effects of different oxidation states and test the dependence of HOCl reactivity on the conserved zinc-binding C52. A C52A mutant models aspects of the Cys-SOH state, having no charge at neutral pH, whereas a C52D mutant may approximate either the Cys-SO^−^ or Cys-SO_2_^−^ state, having a negative charge at neutral pH. In addition, we created DgcZ constructs lacking all cysteines or containing only C52, but these proteins were unstable and unable to be used for *in vitro* experiments (data not shown).

Dimedone is a cyclic diketone that can form stable adducts with cysteine sulfenic acids and is therefore a useful reagent for quantifying cysteine oxidation (53). The reactivity of full-length DgcZ with various concentrations of HOCl was determined through dimedone treatment and slot blotting analysis to monitor formation of Cys-SOH with an antibody that recognizes cysteine-dimedone adducts (Fig. 2B) (53). As the reaction with HOCl is thought to be extremely rapid at neutral pH (approximately 10^9^ M^−1^ s^−1^) (13), we expect that these data reflect reaction endpoints. The wild-type protein contains five cysteines (C52, C85, C223, C230, and C284), and readily forms Cys-SOH in the presence of micromolar HOCl (Fig. 2B). In contrast, the C52D mutant exhibited 5.7-fold less Cys-SOH formation (Fig. 2B). Further characterization using the CZB domain alone (residues 1 to 128) recapitulated this behavior, with a 10 μM concentration of the wild-type protein being half maximally oxidized by 122 μM HOCl and the C52D mutant showing little oxidation even at 500 μM HOCl (Fig. 2C). Using HOCl concentrations above 500 μM reduced the amount of detected Cys-SOH, which we hypothesize is due to overoxidation of C52 to cysteine sulfinate (Cys-SO_2_^−^), as was observed to occur for H. pylori TlpD at similar concentrations (35). In contrast, treatment with equivalent concentrations of H_2_O_2_ produced virtually no Cys-SOH, showing that C52 possesses the ability to discriminate between H_2_O_2_ and HOCl (Fig. 2D) (35). Lastly, preoxidized CZB protein was reduced through the addition of glutathione disulfide, consistent with the reversibility of the Cys-SOH formation (Fig. 2E). These data show that DgcZ has the capacity to react selectively with HOCl through oxidation of C52, can differentiate between HOCl and H_2_O_2_, and can be reduced by reductant systems present in bacteria.

### HOCl oxidation promotes zinc release from the CZB domain.

Zinc is a metal that is essential for many cellular processes, and cells maintain strict control of zinc through the competition of many high-affinity binders that effectively maintain the concentration of free cytosolic Zn^2+^ near zero (54, 55). For example, the E. coli zinc uptake regulator (Zur) protein responds to Zn^2+^ in the subfemtomolar range (56, 57). Hence, the subfemtomolar affinity for Zn^2+^ exhibited by DgcZ (34) positions the enzyme at a threshold necessary to respond to cytosolic zinc. Zinc binding in the CZB domain regulates the enzymatic activity of DgcZ by allosterically inhibiting the productive encounter of two GTP-loaded GGDEF domains to catalyze c-di-GMP (2, 34). Oxidation of zinc-coordinating cysteines by HOCl has been known to promote zinc release (14, 58), which we hypothesized could be a molecular basis by which DgcZ is regulated by HOCl.

Perceiving effects on zinc binding for proteins with such high zinc affinity requires the presence of zinc-binding competitors, as even 10-fold changes will not otherwise substantially alter the zinc-bound ↔ zinc-free equilibrium. Reactions to test CZB zinc lability were performed in PBS buffer, which is both inert to reaction with HOCl and contributes to zinc chelation (55). Relative amounts of soluble Zn^2+^ were monitored with the fluorescent zinc probe zinpyr-1, which has a relatively high affinity for Zn^2+^ and displays a strong increase in fluorescence when zinc bound (35, 59). To assay relative zinc affinity, we competed the wild-type DgcZ protein against the zinpyr-1 probe in the presence of added zinc and observed that DgcZ only slightly diminished the zinc available to the probe (Fig. 2F). To gain insight into the possible effects of cysteine oxidation on zinc binding, we performed equivalent experiments with the C52D mutant and found that the probe’s fluorescence signal was decreased by about half (Fig. 2F).

We next assayed whether HOCl treatment alters CZB zinc binding. HOCl treatment of the wild-type protein showed a bimodal response; zinc was increasingly liberated (made available for the probe to bind) by increasing concentrations of HOCl up to 500 μM (50-fold molar HOCl/DgcZ ratio), and higher HOCl concentrations decreased the amount of zinc available to the probe (Fig. 2G). In contrast, the C52D mutant was unresponsive to HOCl and relinquished little zinc even when treated with millimolar HOCl (Fig. 2G). At 2.5 mM HOCl, the wild-type retention of zinc was approximately equal to that of the C52D mutant (Fig. 2G). Based on the concentrations of HOCl that appear to result in Cys-SOH versus Cys-SO_2_^−^ oxidation states for wild-type DgcZ (Fig. 2C), we interpret these data to indicate that the Cys-SOH state decreases zinc affinity, the Cys-SO_2_^−^ has increased zinc affinity, and that the C52D mutant mimics the increased zinc affinity of the Cys-SO_2_^−^ state (Fig. 2G). The C52D mutant also exhibits higher intrinsic fluorescence and a stronger alpha-helical circular dichroism signature, suggesting that local alterations to the stability of the zinc-binding core induce large-scale changes to the domain’s global structure (Fig. 2H and I).

### Computational dissection of CZB signal transduction.

**FIG 3:**
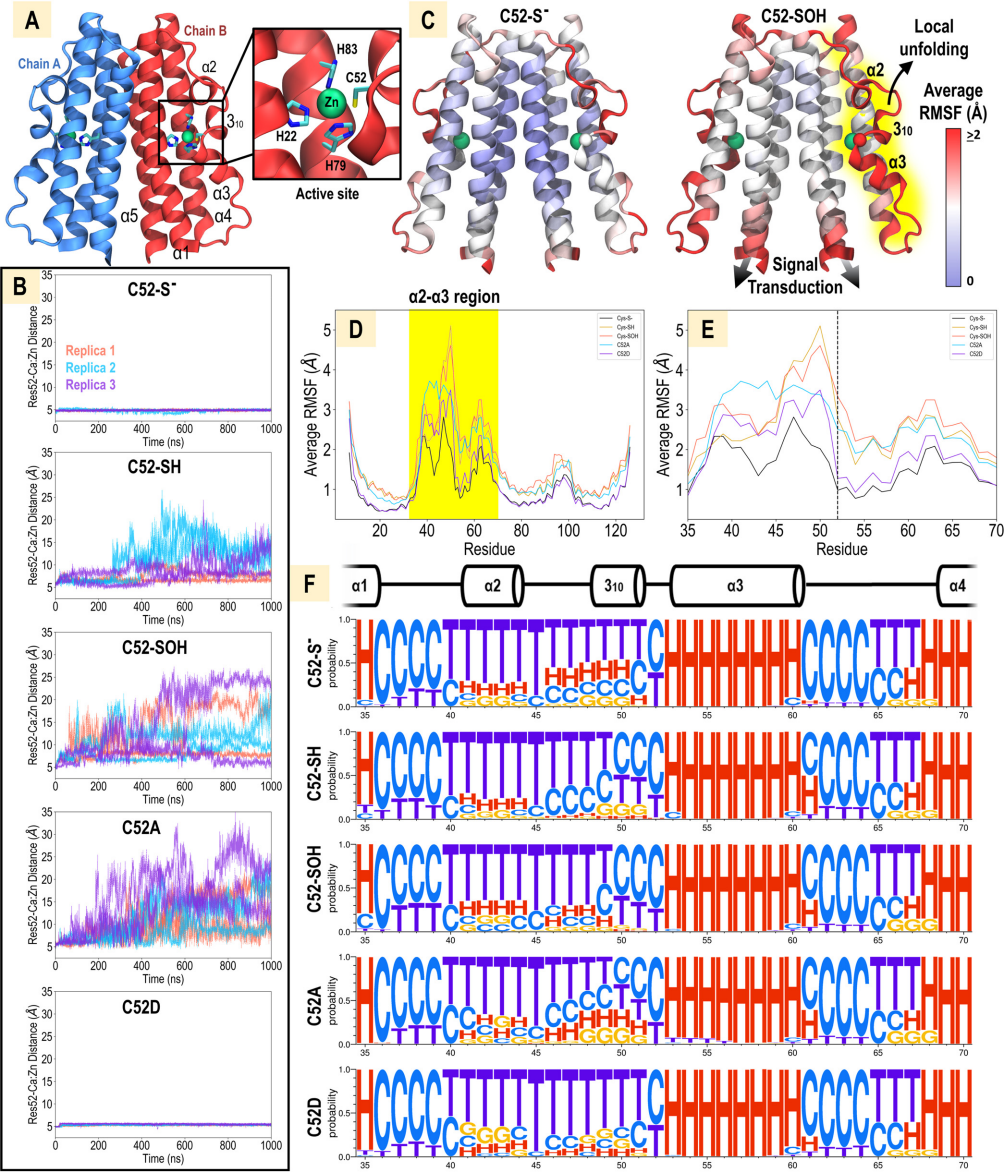
MD simulation of CZB dimer with variable residue 52 moieties. (A) Starting model of the E. coli CZB domain dimer based on crystal structure PDB ID 3T9O (34). (B) Local unfolding events in the α2-α3 region for each model as monitored by the distance between Zn and the residue 52 Cα atoms. Data from three independent simulations are shown (red, blue, purple), with independent active sites from each simulation indicated by solid and dashed lines. (C) RMSFs, averaged across three simulations each, for the C52-S^−^ and C52-SOH models are shown mapped to the CZB structure. The α2-α3 region involved in local unfolding is highlighted in yellow. Black arrows indicate structural changes that may be involved in signal transduction. The zinc and residue 52 positions are represented by spheres. (D) Average RMSF across three simulations for each model is shown, with the α2-α3 region that undergoes local unfolding highlighted in yellow. (E) Close-up view of highlighted region in panel D. Residue 52 is indicated by a black dashed line. (F) Secondary-structure probability profiles, created using Weblogo 3, are shown as determined by Stride (122) (H, α-helix, red; C, coil, blue; T, turn, purple; G, 3_10_-helix, orange). The secondary structure observed in the crystal structure is indicated above the profiles.

We were motivated to learn how the cysteine redox state may alter the zinc-binding core and global CZB structure. Our previous biochemical analysis of TlpD indicated that oxidation can promote local unfolding of the CZB region containing the conserved Cys (α2-α3) (35) (Fig. 3A; Fig. S1B). We wondered if this conformational change could play a role in regulating structurally distant parts of the full-length protein. To address this question, we employed molecular dynamics (MD) simulation to model how HOCl oxidation and zinc release affect the structure of the CZB domain, which contains five α-helices and one 3_10_-helix (Fig. 3A) (PDB ID 3T9O) (34, 60). We examined three redox states of the conserved zinc-binding C52, namely, C52-S^−^ (native unreacted thiolate state when bound to zinc), C52-SH, and C52-SOH (product of HOCl reaction), as well as two mutations, C52A and C52D. For each state, triplicate simulations were performed for a duration of 1 μs each, allowing six views of the active site (Movies S1A to S1E).

Our simulations revealed a loss of coordination between residue 52 and the zinc atom within the Cys-SH, Cys-SOH, and C52A simulations, while the pair maintain tight interactions throughout the C52D and Cys-S^−^ simulations (Fig. 3B). This loss of coordination has two principal effects. First, it leads to increased zinc lability and release from the active site, as demonstrated by a corresponding increase in the distance between zinc and the zinc-binding core residue His83 (Fig. S2A to E). Second, it markedly affects the conformational flexibility of the entire α2-α3 segment (approximately residues 39 to 65) surrounding residue 52. This observation is in line with previous crystallographic and biochemical data suggesting that α2-α3 becomes disordered upon disruptions to the zinc-thiolate interaction (34, 35). This effect is illustrated by the stark increase in average root mean square fluctuation (RMSF) of this region for Cys-SOH compared to Cys-S^−^ (Fig. 3C; Fig. S2F). Interestingly, the increased dynamics of the C52 position and α2-α3 segment are propagated to the N and C termini, which connect to the downstream- and upstream-regulated protein domains, respectively (Fig. 3C, signal transduction arrows). Comparison of average RMSFs across all systems reveals that the Cys-SH, C52A, and Cys-SOH states, which disrupt zinc-cysteine interactions, display increased global dynamics (Fig. 3D and E). Consistent with the trends of RMSF, an analysis of average secondary structure probability of the α2-α3 region showed that more random coil is observed at the 3_10_-helix for C52A, Cys-SH, and Cys-SOH, reflecting their increased dynamics and propensity to undergo local unfolding, while less random coil is observed for Cys-S^−^ and the C52D mutant, reflecting those models’ stability and generally static nature (Fig. 3F). These data indicate that changes to the oxidation state of C52 can induce large-scale alterations to CZB structure and dynamics.

10.1128/mBio.00173-21.2 FIG S2 Molecular dynamic simulation shows structural impacts of C52 redox state and mutation. (A to E) Zinc release events monitored by distance between the zinc atom and the zinc-coordinating residue H83. (F) Difference in average RMSF between Cys-SOH and Cys-S^−^ simulations. Download FIG S2, TIF file, 2.8 MB. Copyright © 2021 Perkins et al. 2021 Perkins et al. https://creativecommons.org/licenses/by/4.0/ This content is distributed under the terms of the Creative Commons Attribution 4.0 International license.

To further understand how HOCl may promote CZB local unfolding and signal transduction, we performed quantum mechanics (QM) calculations quantifying the energetic tendency for different cysteine redox states to be displaced from the CZB domain by either water or the HOCl ligand. Using the core of the CZB domain in E. coli DgcZ (34) as a model system, we examined the ligand exchange equilibria at three redox and protonation sulfur states, namely, CH_3_S (H), CH_3_SO (H), and CH_3_SO_2_ (H) (Table 1). Consistent with results from our MD simulations, our QM calculations indicate that in all but one scenario, the protonated sulfur states are more likely to be displaced from the zinc than the deprotonated states. Based on approximate pKa’s of Cys-SH (pKa, ∼8.6) (61), Cys-SOH (pKa, ∼6.3 to 12.5) (62, 63), and Cys-SO_2_H (pKa, ∼1.8) (64), we suggest the following model at neutral cytosolic pH for the relative tendency of discrete cysteine redox states to be displaced from the zinc complex. Prior to oxidation, the deprotonated Cys-S^–^ state is energetically favored to remain associated with the zinc complex (Δ*G* for displacing CH_3_S^–^ = 16.9 kcal/mol by H_2_O and 7.2 kcal/mol by HOCl). However, upon oxidation by HOCl, the protonated Cys-SOH state is more readily displaced from the complex (Δ*G* for displacing CH_3_SOH = 1.4 kcal/mol by H_2_O and −0.4 kcal/mol by HOCl), and even the deprotonated Cys-SO^–^ state is destabilized compared to the unreacted Cys-S^−^ state (Δ*G* for displacing CH_3_SO^–^ = 3.6 kcal/mol by H_2_O and −2.5 kcal/mol by HOCl). Following overoxidation, the Cys-SO_2_^–^ protonation state predominates and is less favored than the Cys-SO^–^ state to dissociate from the complex (Δ*G* for displacing CH_3_SO_2_^–^ = 9.1 kcal/mol by H_2_O and 8.7 kcal/mol by HOCl).

**TABLE 1:**
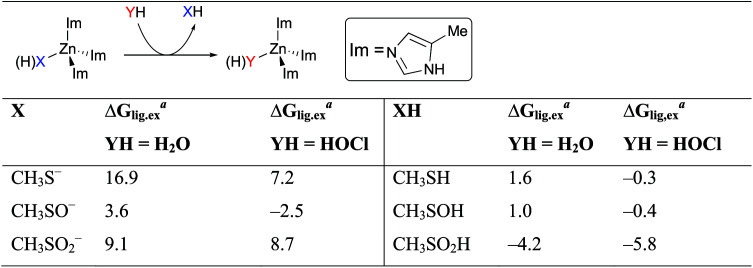
Ligand exchange equilibria depicting the energetic tendency for the sulfur ligand across protonation and oxidation states to dissociate from a model Zn^2+^ complex

a Energies were calculated at 298 K and 1.0 atm and are reported in kcal/mol. Energies reflect the exchange between various sulfur ligands of the zinc-binding core (X) to be displaced by ligands of different protonated states (Y).

### HOCl relieves Zn-mediated inhibition of DgcZ enzymatic activity.

**FIG 4:**
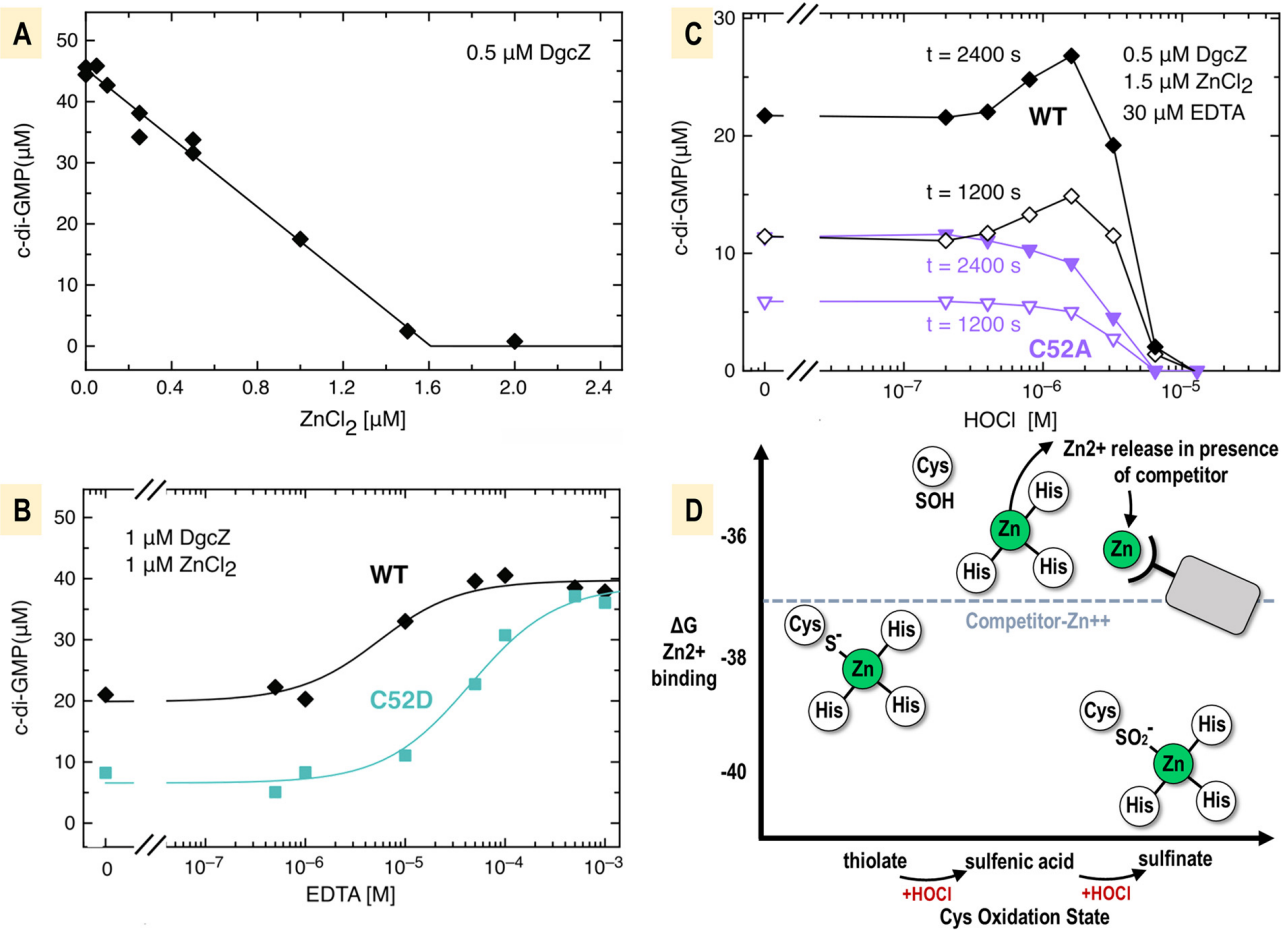
Relief of zinc-mediated inhibition of DgcZ activity by HOCl. DgcZ-catalyzed c-di-GMP synthesis was assayed by FPLC nucleotide concentration determination at saturating GTP (500 μM for panels A and B and 300 μM for panel C) and the indicated enzyme concentrations. Incubation times were 1,800 s and 1,037 s for panels A and B, respectively. (A) Zinc titration of wild-type DgcZ demonstrating linear decrease of c-di-GMP production (solid line) consistent with high-affinity binding of the zinc inhibitor. (B) EDTA titration relieves zinc-mediated enzyme inhibition. Solid lines represent the fit with a two-state model. For the C52D mutant, about a 10-fold-higher EDTA concentration is required for the half-maximal effect, indicating tighter zinc binding of the mutant than of the wild type (WT). (C) HOCl titration in the presence of EDTA competition for zinc shows relief of DgcZ inhibition at low HOCl concentration (≤2 μM) for the wild type but not for the C52A mutant. The C52A mutant demonstrates the effect of dose-dependent inactivation, seen also for the wild type at higher HOCl concentrations. Incubation times were as indicated. (D) Energetic map for the effect of HOCl oxidation on DgcZ zinc lability based on calculations of wild-type, C52A (34) and C52D zinc affinities (Δ*G* in units of gas constant [R] × temperature [T]). Zinc competitors are required to establish a threshold (dashed line) for cysteine oxidation to be able to alter the CZB zinc-binding equilibrium. All data values are available in Data Set S1A in the supplemental material.

Although we had established that oxidation of DgcZ by HOCl promotes zinc release, we had yet to determine whether enzyme activity is regulated in this manner. Therefore, we performed activity assays measuring DgcZ production of c-di-GMP under various conditions. Titration of ZnCl_2_ results in a linear decrease of enzymatic activity as measured by the concentration of the c-di-GMP product after a defined incubation time (Fig. 4A). Under these conditions, a superstoichiometric amount of ZnCl_2_ is required for complete inhibition, likely due to Zn^2+^ complexation with phosphate from the buffer. Zinc-mediated inhibition of DgcZ can be relieved by the addition of a zinc chelator like EDTA that competes for Zn^2+^ (34). Partially zinc-loaded wild-type DgcZ shows a clear activation by EDTA which can be fit well with a two-state model. Under the same conditions, the C52D mutant shows considerably lower activity without EDTA but converges to the same maximal value at high EDTA (Fig. 4B). The data conform to a two-state model, but the inflection point occurs at approximately 10-fold higher EDTA, consistent with Zn^2+^ affinity being higher for the C52D mutant than for the wild-type protein (Fig. 4B).

We performed a titration of HOCl in the presence of EDTA under conditions where the available Zn^2+^ was estimated to be shared about equally between DgcZ and EDTA (Fig. 4C). Under these conditions, HOCl treatment increased DgcZ activity for HOCl of ≤2 μM (4-fold molar HOCl/DgcZ ratio), whereas larger concentrations induced a negative effect (Fig. 4C). Experiments with the C52A mutant showed no activation by HOCl but did show inactivation at higher HOCl concentrations similar to that of the wild-type protein (Fig. 4C). This leads us to conclude that the activating effect of HOCl seen for wild-type DgcZ is due to specific oxidation of C52 to Cys-SOH and concomitant release of Zn^2+^ by the CZB domain (Fig. 4D). We had been interested to learn if specific overoxidation of C52 to Cys-SO_2_^−^ might also serve a regulatory purpose, potentially as a way for bacteria to perceive high concentrations of HOCl. However, both the wild type and the C52A mutant were inhibited at higher HOCl concentrations, suggesting that the effects are due to nonspecific protein oxidation. The ability of DgcZ to be oxidized by HOCl and to regulate c-di-GMP production provides a molecular basis for our observations of biofilm regulation in response to exogenous HOCl (Fig. 1).

### C52 is required for regulation of E. coli biofilm formation in response to HOCl.

Limited knowledge of the molecular basis of CZB ligand sensing has restricted understanding of the biological roles of CZB domains, and most studies so far have relied on interpreting the phenotypes of strains harboring full genetic knockouts (30, 34–36, 65) rather than dissecting CZB function at the individual amino acid level. Although the high conservation of the CZB zinc-binding cysteine has been documented in other studies (34, 35), its importance for function has never been demonstrated *in vivo*. We were interested to test the requirement for C52 for mediating biofilm responses to HOCl in E. coli and to further explore how discrete cysteine oxidation states of DgcZ influence cellular c-di-GMP signaling. However, a practical barrier to performing these experiments in bacteria is the transient nature of the Cys-SOH reaction intermediate, which can be reduced or further oxidized to Cys-SO_2_^−^ (66, 67). Our biochemical and computational characterization of DgcZ cysteine point mutants provided a strategy to circumvent these difficulties, as these redox states can be stably approximated by the C52A and C52D mutants. We therefore created two new E. coli strains through seamless CRISPR gene editing that contain *dgcZ* chromosomal point mutants to express *dgcZ^C52A^* or *dgcZ^C52D^* under native gene regulation.

**FIG 5:**
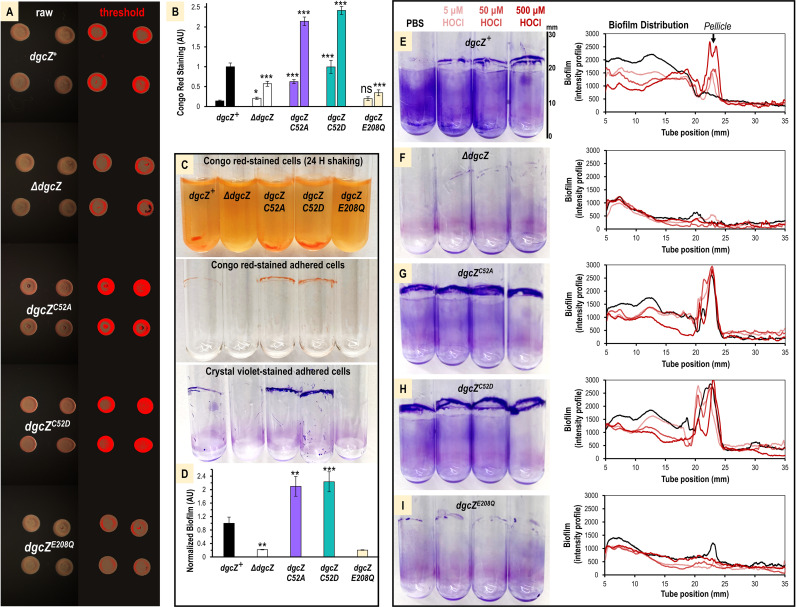
Role of conserved C52 in HOCl sensing and biofilm distribution. (A) Representative images showing growth of cells expressing functional DgcZ (*dgcZ^+^*), lacking DgcZ (Δ*dgcZ*), containing cysteine point mutants (*dgcZ^C52A^*, *dgcZ^C52D^*), or a form unable to catalyze c-di-GMP (*dgcZ^E208Q^*) on Congo red-LB agar plates after 24 h at 25°C. Plates were inoculated with 2 μl of liquid cultures grown overnight in LB. Raw images are shown on the left, and the color-thresholded versions are shown on the right. (B) Quantification of Congo red dye uptake from experiments described for panel A (right column) and equivalent experiments inoculated with mid-log exponential cells at an *A*_600_ of 0.5 (left column) (*n* = 12). (C) Comparison of Congo red dye uptake and biofilm formation after 24 h of static growth at 30°C. (D) Quantification of biofilm experiments described for panel C by crystal violet staining (*n* = 10). (E to I) Biofilm distribution and pellicle formation is shown for cells treated with PBS (black), 5 μM HOCl (light red), 50 μM HOCl (medium red), or 500 μM HOCl (dark red) and grown for 24 h shaking at 30°C. Representative images of crystal violet-stained tubes are shown on the left, and the average biofilm density along the vertical length of the tubes is shown as an intensity profile on the right (*n* = 3). Quantification of data including error bars is shown in Fig. S3C in the supplemental material. All data values are available in Data Set S1A. *P* values from paired Student *t* tests are shown between treated and untreated controls. ns, not significant (*P* > 0.05); *, *P* < 0.05; **, *P* < 0.01; ***, *P* < 0.001.

DgcZ production of c-di-GMP is known to regulate synthesis of the biofilm polymer poly-N-acetylglucosamine (poly-GlcNAc), which can be visualized with Congo red dye (34, 43). We assessed the staining of E. coli strains on LB agar plates containing Congo red, including as a control a strain expressing an enzymatically inactivated *dgcZ^E208Q^* mutant (34). Under these conditions, we found that the wild type exhibited a modest amount of Congo red binding, the *dgcZ^C52A^* and *dgcZ^C52D^* mutants showed increased binding, and the Δ*dgcZ* and *dgcZ^E208Q^* mutants showed low dye binding (Fig. 5A). To quantify dye binding in these assays, images of colonies were color thresholded, and the fractions of red pixels (dye-containing cells) out of the total pixels (e.g., brown [non-dye containing cells] and red) were normalized to those of the wild-type strain (Fig. 5B). For plates inoculated with cells from mid-log exponential cultures, the *dgcZ^C52A^* and *dgcZ^C52D^* mutants showed 4.4- and 7.0-fold greater dye binding, respectively, than the *dgcZ^+^* strain (Fig. 5B, left column). Plates inoculated with cells from established overnight cultures also showed elevated Congo red binding by *dgcZ^C52A^* and *dgcZ^C52D^* mutants, 2.1 and 2.4-fold higher than the wild type, respectively, with the *dgcZ^+^* strain showing a moderate amount and Δ*dgcZ* and *dgcZ^E208Q^* mutants showing lowered binding (Fig. 5B, right column).

10.1128/mBio.00173-21.3 FIG S3 Biofilm quantification for wild-type and mutant E. coli strains. (A) Biofilm formation of wild-type and mutant lines after 24 h of static growth in LB at 30°C (*n* = 4). (B) Biofilm formation for cells as described for panel A but exposed to point source treatments, as indicated (*n* = 6). (C) Pellicle formation quantified from Fig. 5E to I for the region spanning pixels 2000 to 25000 for wild-type (black), *ΔdgcZ* (gray), *dgcZ^C52A^* (violet), *dgcZ^C52D^* (teal), and *dgcZ^E208Q^* (light yellow) strains (*n* = 3). (D) Biofilm formation after treatments with ZnSO_4_ and incubation with gentle rocking for 24 h at 30°C (*n* = 3). Data points are sample means, and error bars are standard errors of the mean. *P* values from paired Student *t* tests are shown between *dgcZ^+^* and mutant strains with *P* values of >0.05 noted as not significant (ns). *, *P* < 0.05; **, *P* < 0.01; ***, *P* < 0.001. Download FIG S3, TIF file, 1.4 MB. Copyright © 2021 Perkins et al. 2021 Perkins et al. https://creativecommons.org/licenses/by/4.0/ This content is distributed under the terms of the Creative Commons Attribution 4.0 International license.

That both the *dgcZ^C52A^* and *dgcZ^C52D^* mutants appeared to produce increased poly-GlcNAc ran counter to our prediction based on *in vitro* analyses, in which we expected the differences in zinc-mediated inhibition of c-di-GMP synthesis to result in increased and decreased production of poly-GlcNAc, respectively. We also found both *dgcZ^C52A^* and *dgcZ^C52D^* mutants to have increased biofilm formation when grown in static liquid cultures (Fig. S3A) and rocking liquid cultures (Fig. 5C and D). But, in a point source assay, the cellular behavior we expected based on *in vitro* assays with the recombinant protein was somewhat recapitulated, with wild-type biofilm formation increased by HOCl, the *dgcZ^C52A^* mutant being unresponsive, and the *dgcZ^C52D^* mutant showing decreased biofilm (Fig. S3B). Therefore, behavior of the *dgcZ^C52A^* mutant (Cys-SOH mimic) was consistent with our biochemical and computational modeling, whereas the behavior of the *dgcZ^C52D^* mutant (Cys-SO_2_^−^ mimic) was more difficult to interpret.

In rocking cultures, we frequently observed that biofilm did not occur evenly and a robust pellicle (68, 69) formed at the liquid-air interface (Fig. 5C). Experiments visualizing biofilm formation in proximity to an HOCl microgradient had suggested that biofilm distribution might be regulated by DgcZ (Fig. 1I). We therefore tested if pellicle formation was altered by HOCl and whether this required C52 *in vivo*. Cultures of *dgcZ^+^* cells treated with increasing concentrations of HOCl diluted in PBS buffer showed a dose-dependent increase in pellicle formation over those with PBS treatment alone (Fig. 5E). Equivalent experiments with *dgcZ^C52A^* and *dgcZ^C52D^* mutants showed robust pellicle formation regardless of HOCl treatments, and Δ*dgcZ* and *dgcZ^E208Q^* mutants exhibited low biofilm and little change in pellicle formation (Fig. 5F to I; Fig. S3C). Similar biofilm experiments with addition of exogenous zinc partially recapitulated *in vitro* observations of differences in zinc-mediated inhibition of DgcZ c-di-GMP of the wild type and cysteine mutants, with addition of 50 μM zinc decreasing biofilm for *dgcZ^+^* and *dgcZ^C52D^* strains and the *dgcZ^C52A^* mutant being less sensitive to zinc inhibition (Fig. S3D). Based on these data, we conclude that DgcZ utilizes C52 for sensing HOCl to regulate poly-GlcNAc-dependent biofilm formation and biofilm distribution.

### Architectures, conservation, and biological distribution of CZB-containing proteins.

Our initial research question, motivated by our earlier discovery of an H. pylori CZB-containing chemoreceptor that mediates attraction to HOCl sources (35), was whether CZB domains could be a previously unappreciated and widespread mechanism by which host-associated bacteria perceive inflammation by acting as HOCl sensors. We next expanded our analysis by performing a bioinformatics survey of CZB domain sequences to quantify the prevalence and biological distribution of CZB-containing proteins.

**FIG 6:**
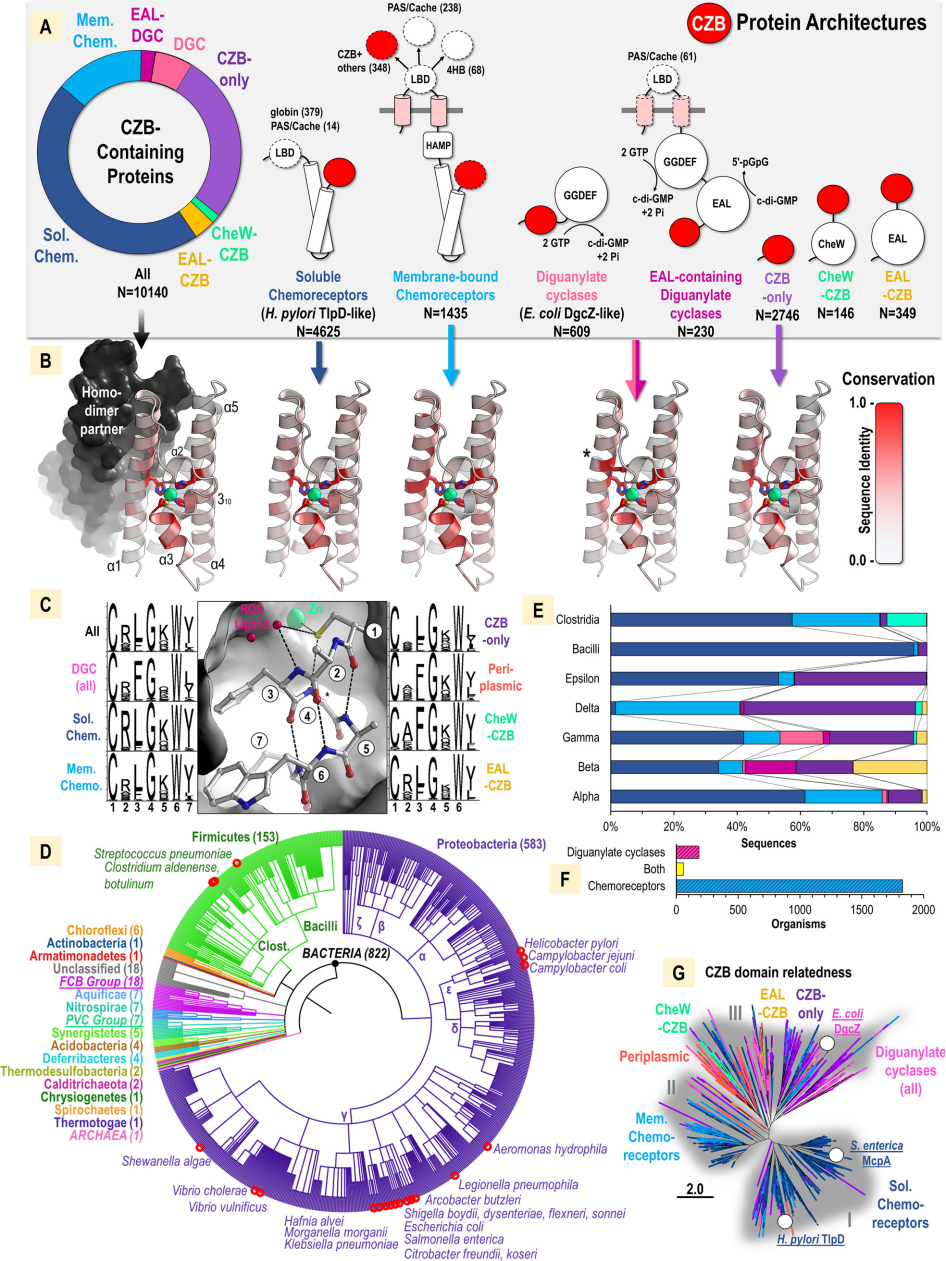
Amino acid conservation and biological distribution of CZB domains. (A) Prevalence of different domain architectures of CZB domain-containing proteins, colored as follows: soluble chemoreceptors, dark blue; membrane-bound chemoreceptors, light blue; diguanylate cyclases (DGC), light pink; EAL-containing diguanylate cyclases, dark pink; EAL-CZB, gold; CheW-CZB, mint green; CZB only, purple. Diagrams of protein domain architecture show the CZB domain as a red circle with the N terminus represented by a short, curved line. Membrane-spanning regions are indicated in pink. Structural features that are variable within a subgroup are represented by dashed lines, with variations in ligand-binding domain (LBD) quantified in parentheses. Observed domains include four-helix bundle (4HB), globin, Pas/Cache (and tandem PAS/dCache), histidine kinases–adenylate cyclases–methyl-accepting proteins–phosphatase (HAMP), methyl-accepting chemotaxis proteins (MCP, here referred to as chemoreceptors), CheW-like, GGDEF, and EAL. (B) Amino acid conservation of CZB subgroups mapped onto the crystal structure of E. coli DgcZ CZB (PDB 3T9O). DGCs with and without EAL domains (light and dark pink) were similar and are mapped as a single group that shows an additional conserved Trp (asterisk). (C) Amino acid conservation of the α3 CZB motif among CZB protein subgroups. An additional “periplasmic” group which comprises putative periplasmic CZBs from the N-terminal region of membrane-bound chemoreceptors (orange) is included. The predicted binding site for reactive oxygen species ligands, such as HOCl, is approximated by two bound water molecules in the CZB structure (PDB 3T9O) and indicated as “ROS ligand” in pink. (D) Phylogenetic tree showing the biological distribution of CZB domains colored by phyla. The number of organisms identified to the species level in each group is noted in parentheses. The classes *Firmicutes* and *Proteobacteria* are indicated. Bacteria associated with causing disease in humans are indicated by red circles. (E) CZB subgroups found in classes *Firmicutes* and *Proteobacteria*. The coloring scheme is as described for panel A, and the subgroups are ordered left to right as soluble chemoreceptors, membrane-bound chemoreceptors, diguanylate cyclases, EAL-containing diguanylate cyclases, CZB only, CheW-CZB, and EAL-CZB. (F) Quantification of organisms that at the species level contain only a CZB-containing chemoreceptor (light and dark blue), only a diguanylate cyclase (light and dark pink), or both (yellow). (G) Relatedness tree of CZB domains from subgroups, with clusters I to III shaded in gray.

To create a database of all currently available CZB-containing proteins, we performed iterative searches with the Basic Local Alignment Search Tool (BLAST) (70) for CZB domain sequences in the nonredundant protein database, which resulted in 10,140 unique sequences (Data Set S1C). Most CZB-containing protein sequences contain multiple protein domains, so to understand the major cellular pathways regulated by CZB domains, we further categorized and quantified sequences according to protein domain architecture. We found that CZB-containing proteins can be divided into seven subgroups based on domain similarity, with the majority of sequences involved in two biological outputs, namely, chemotaxis or c-di-GMP metabolism (Fig. 6A and B). Some sequences also appear to contain only a CZB domain with no other detectable protein domain sequence signature (27.1%), although some of these may represent incomplete sequences or annotations. The most common subgroup consists of soluble CZB-regulated chemoreceptors similar in structure to H. pylori TlpD (45.6%), which we refer to here as “TlpD-like.” CZB-regulated nucleotide cyclases, including E. coli DgcZ, account for a smaller but widespread fraction of sequences (6.0%), which we refer to as “DgcZ-like” (Table 2). Less common, but involved in functionally related processes, are CZB-regulated chemotaxis W (CheW, 1.4%) and Glu-Ala-Leu (EAL) (3.4%) proteins that transduce chemoreceptor signals and degrade c-di-GMP, respectively. Nearly all CZB sequences are predicted to be cytosolic, with only 384 putative periplasmic CZB sequences (approximately 3.9%) identified.

**TABLE 2:** Bacteria that possess DgcZ-like proteins^*a*^

Genus/species^*b*^	Accession no./locus tag	Host association
*Achromobacter* sp. ATCC 35328	CUK19701.1	Yes^*c*^
**Citrobacter freundii**	WP_071685553.1	Yes
**Citrobacter koseri**	WP_047457159.1	Yes
Citrobacter pasteurii	WP_121584627.1	Yes
Citrobacter portucalensis	WP_079934014.1	Yes^*c*^
*Curvibacter* sp. GWA2_64_110	OGP03434.1	Yes^*c*^
Dyella ginsengisoli	WP_017462428.1	No
**Escherichia coli**	WP_000592841.1	Yes
Hyphomonas adhaerens	WP_162177456.1	No
*Hyphomonas* sp. CY54-11-8	WP_051599791.1	No
*Hyphomonas* sp. GM-8P	WP_112073350.1	No
*Hyphomonas* sp. ND6WE1B	WP_065383192.1	No
**Legionella cincinnatiensis**	WP_065240083.1	Yes
Legionella gratiana	WP_065231755.1	Yes
**Legionella hackeliae**	WP_045105921.1	Yes
**Legionella pneumophila**	GAN16124.1	Yes
Legionella santicrucis	WP_065236300.1	Yes
Oceanospirillum linum	OOV86010.1	Yes^*c*^
Oceanospirillum maris	WP_028304308.1	Yes^*c*^
Oceanospirillum sanctuarii	WP_086478956.1	Yes^*c*^
Oleiagrimonas soli	WP_052394942.1	No
*Oleiagrimonas* sp. MCCC 1A03011	WP_113063355.1	No
*Parvibaculum* sp. HXT-9	WP_152215867.1	No
*Rhodanobacter* sp. SCN 68-63	ODV15579.1	No
**Shigella boydii**	EAA4815907.1	Yes
**Shigella dysenteriae**	WP_000592774.1	Yes
**Shigella flexneri K-315**	EIQ21590.1	Yes
**Shigella sonnei**	CSF33682.1	Yes
**Streptococcus pneumoniae**	VTQ33263.1	Yes
Sulfurimonas gotlandica	WP_008337708.1	No
*Sulfurimonas* sp. GYSZ_1	WP_152307624.1	No
*Thiotrichales* bacterium 12-47-6	OZB86354.1	No
*Thiotrichales* bacterium 32-46-8	OYX07815.1	No
Tistlia consotensis	WP_085120475.1	No

a Protein architecture consists of an N-terminal CZB domain and a C-terminal GGDEF domain.

b Human pathogens are indicated in bold.

c Bacteria of this genus are known to be host associated, but this has not been directly determined for this species.

Protein structure-function relationships can be revealed through analyses of conservation patterns to learn what parts of the protein are indispensable for function across divergent homologues (71–73). Commonalities in amino acid conservation between distantly related CZB sequences could point toward general functions of CZB domains, while differences between subgroups could indicate evolutionary tuning to optimize ligand sensing and signal transduction in specific settings. In addition to the ubiquitous 3His/1Cys zinc-binding motif, two regions of global conservation across all CZB domains were revealed (Fig. 6B). First, the N-terminal α1-helix exhibits a modest degree of conservation, with 10 positions that have sequence identity conservation in the range of 20% to 100%. This region (residues 1 to 30) constitutes a large portion of the homodimer interface (384 Å^2^ of 1,950 Å^2^ total) that forms a 2-fold symmetr*y* axis, with residues packing against their homodimer counterpart. Second, in addition to the universally conserved zinc-binding Cys, many residues of the α3 region exhibit a high degree of conservation (Fig. 6B). This pattern of conservation in the α1 and α3 regions occurs across all CZB subgroups, suggesting that these two regions are of universal importance for CZB function. One additional site of high conservation occurs in the diguanylate cyclase subgroup, where a Trp, which resides three residues downstream of the conserved zinc-binding His22, packs into the protein core against the zinc-binding site (Fig. 6B, indicated by an asterisk).

The high conservation of the α3 region could relate to CZB signal transduction, as we showed that this region is involved in redox-stimulated conformational changes (Fig. 3). To further investigate the conservation of the α3 region, seqLogo plots were generated for the seven-residue motif containing the conserved Cys across all CZB sequences and individual CZB architecture subgroups (Fig. 6C). By studying the position and interactions of each residue in the E. coli DgcZ CZB structure, putative roles and rationales for conservation were inferred for each amino acid site as follows. Position 1 is approximately 100% conserved as a Cys, reflecting its absolute requirement for function. The Cys forms a lid for the zinc-binding core, increases zinc affinity by an order of magnitude (34), and can serve as an HOCl sensor through direct oxidation (35). Positions 2 and 5 are conserved as residues that contain either a hydrophilic side chain or a small hydrophobic side chain that permits exposure to solvent. Positions 3, 6, and 7 are conserved as bulky hydrophobic side chains that are buried in the protein core and provide a thermodynamic driving force for the proper folding of the motif. Position 4 is almost universally conserved as a Gly, because a C_β_ atom would clash with the carboxyl oxygen of a position three residues upstream of the Cys in the 3_10_-helix, and the position does not adopt phi-psi angles that are Gly specific (φ = 122.3°, ψ = 114.9°). A site of variability between subgroups is position 2, which is enriched as Arg especially in soluble chemoreceptors but also in membrane-bound chemoreceptors, diguanylate cyclases, and EAL-CZBs (Fig. 6C). However, position 2 is either poorly conserved or conserved as an Ala for the CZB-only, periplasmic, and CheW-CZB subgroups.

To obtain insight into the biological factors that drive CZB evolution, we assessed the phylogenetic distribution of CZB domains. This revealed a total of 822 unique organisms for which phylogenetic classification and annotation were available down to the species level. This analysis shows that CZB domains are found in diverse phyla and essentially all CZB sequences are bacterial (Fig. 6D). A single eukaryotic sequence from the nematode Diploscapter pachys was nearly identical to a sequence from Pseudomonas and, therefore, likely contamination. A single archaeon sequence from “*Candidatus* Woesearchaeota” shows 35% sequence similarity to sequences from the bacterial genus *Sulfurimonas* and may represent a case of horizontal gene transfer. In total, CZB domains were identified in 21 bacterial phyla and 6 candidate phyla (Fig. 6D). An evolutionary divergence tree for species that contain CZB proteins suggests that they may have arisen in bacteria more than 4 billion years ago (Fig. S4) (74).

10.1128/mBio.00173-21.4 FIG S4 Divergence tree for CZB-containing species. Scale reflects billions of years ago (BYA) from the present day (circle exterior) dating back to the bacterial last universal common ancestor (LUCA; red circle). The approximate origins of eukaryotes and animals are noted (black arrow). Download FIG S4, TIF file, 2.8 MB. Copyright © 2021 Perkins et al. 2021 Perkins et al. https://creativecommons.org/licenses/by/4.0/ This content is distributed under the terms of the Creative Commons Attribution 4.0 International license.

10.1128/mBio.00173-21.5 MOVIE S1A Molecular dynamics simulation of CZB oxidation and mutants. (A) Representative molecular dynamics simulation of wild-type CZB in the C52-S^−^ state. Download MOVIE S1A, MPG file, 7.6 MB. Copyright © 2021 Perkins et al. 2021 Perkins et al. https://creativecommons.org/licenses/by/4.0/ This content is distributed under the terms of the Creative Commons Attribution 4.0 International license.

10.1128/mBio.00173-21.6 MOVIE S1B Molecular dynamics simulation of CZB oxidation and mutants. (B) Representative molecular dynamics simulation of wild-type CZB in the C52-SH state. Download MOVIE S1B, MPG file, 7.9 MB. Copyright © 2021 Perkins et al. 2021 Perkins et al. https://creativecommons.org/licenses/by/4.0/ This content is distributed under the terms of the Creative Commons Attribution 4.0 International license.

10.1128/mBio.00173-21.7 MOVIE S1C Molecular dynamics simulation of CZB oxidation and mutants. (C) Representative molecular dynamics simulation of wild-type CZB in the C52-SOH state. Download MOVIE S1C, MPG file, 8.3 MB. Copyright © 2021 Perkins et al. 2021 Perkins et al. https://creativecommons.org/licenses/by/4.0/ This content is distributed under the terms of the Creative Commons Attribution 4.0 International license.

10.1128/mBio.00173-21.8 MOVIE S1D Molecular dynamics simulation of CZB oxidation and mutants. (D) Representative molecular dynamics simulation of CZB-C52A. Download MOVIE S1D, MPG file, 8.2 MB. Copyright © 2021 Perkins et al. 2021 Perkins et al. https://creativecommons.org/licenses/by/4.0/ This content is distributed under the terms of the Creative Commons Attribution 4.0 International license.

10.1128/mBio.00173-21.9 MOVIE S1E Molecular dynamics simulation of CZB oxidation and mutants. (E) Representative molecular dynamics simulation of CZB-C52D. Download MOVIE S1E, MPG file, 7.7 MB. Copyright © 2021 Perkins et al. 2021 Perkins et al. https://creativecommons.org/licenses/by/4.0/ This content is distributed under the terms of the Creative Commons Attribution 4.0 International license.

In terms of currently available sequence data, most CZB proteins are found in *Firmicutes* and *Proteobacteria*, phyla that contain many host-associated species, and *Gammaproteobacteria* account for approximately one-third of all known CZB sequences (Fig. 6D). CZB domains were identified in 23 bacterial species associated with human diseases, including many enteric pathogens (Fig. 6D, red circles; Table 2). There exist considerable differences among the *Proteobacteria* and *Firmicutes* classes in the prevalence of CZB protein architectures (Fig. 6E). Regarding subgroups involved in chemotaxis, soluble chemoreceptors make up a sizable fraction in these classes, except for Deltaproteobacteria, in which they are nearly absent, and CheW-CZBs are most abundant in *Clostridia*. Diguanylate cyclases and EAL-CZBs are restricted to *Alpha*-, *Beta*-, *Gamma*-, and Deltaproteobacteria, suggesting that CZBs are important regulators of c-di-GMP signaling for these bacteria. At the species level, we were intrigued to find that bacteria seem to contain either CZB-regulated chemoreceptors or CZB-regulated diguanylate cyclases, but not both (Fig. 6F).

CZB domains may have become honed over time to perform signaling and regulation in specific contexts. We examined whether or not the similarity of CZB domain sequences corresponds to their function (i.e., domain structure). A relatedness tree for full-length CZB amino acid sequences provides some evidence of this. Approximately three main clusters exist: cluster I is dominated by C-terminal soluble chemoreceptor CZBs, including H. pylori TlpD and S. enterica methyl-accepting chemotaxis protein A (McpA), cluster II is dominated by C-terminal membrane-bound chemoreceptor CZBs, and cluster III is more variable and contains diguanylate cyclases, including E. coli DgcZ, periplasmic (N-terminal chemoreceptor CZBs), CheW-CZB, EAL-CZB, and CZB-only subgroups (Fig. 1G). Of these, we have now demonstrated that clusters I and III both contain representative CZB domains that are HOCl sensors, supporting that HOCl sensing is retained across distantly related CZB homologues from host-associated bacteria.

## DISCUSSION

CZB domains are a class of sensors that have remained enigmatic despite their prevalence among diguanylate cyclases and chemoreceptors in diverse bacterial phyla (Fig. 6) (33). In fact, CZB domains are the most common C-terminal regulatory domains of cytoplasmic chemoreceptors (75). Our investigation has yielded a new rationale for the widespread conservation of CZB domains in bacteria: they are versatile sensory apparatuses with the potential to perceive and integrate information from multiple effectors through zinc binding. We first consider the function of HOCl sensing by CZB domains, which occurs through direct oxidation of the zinc-binding cysteine. This response may be most relevant for bacteria that colonize animals and are exposed to high concentrations of HOCl through the neutrophilic respiratory burst, but these domains may also function as monitors of other exogenous effectors or of intracellular processes that alter zinc homeostasis.

### Bacterial lifestyle decisions based on specific environmental oxidants.

Earlier work has elucidated mechanisms by which bacteria sense and tolerate HOCl in their environment, with HOCl-sensitive transcription factors orchestrating a sophisticated network of proteins that eliminate oxidants and repair oxidative damage (17–23). Here, we add to this knowledge by presenting evidence that CZB domains are a prevalent HOCl sensory system used by bacteria to regulate the decision between motility and sessility, implicating these proteins as important players in bacterial coexistence with inflammation. Our findings suggest that host-associated bacteria may regard HOCl not only as a toxic oxidant but also as a reporter on host inflammation that can inform bacterial localization.

**FIG 7:**
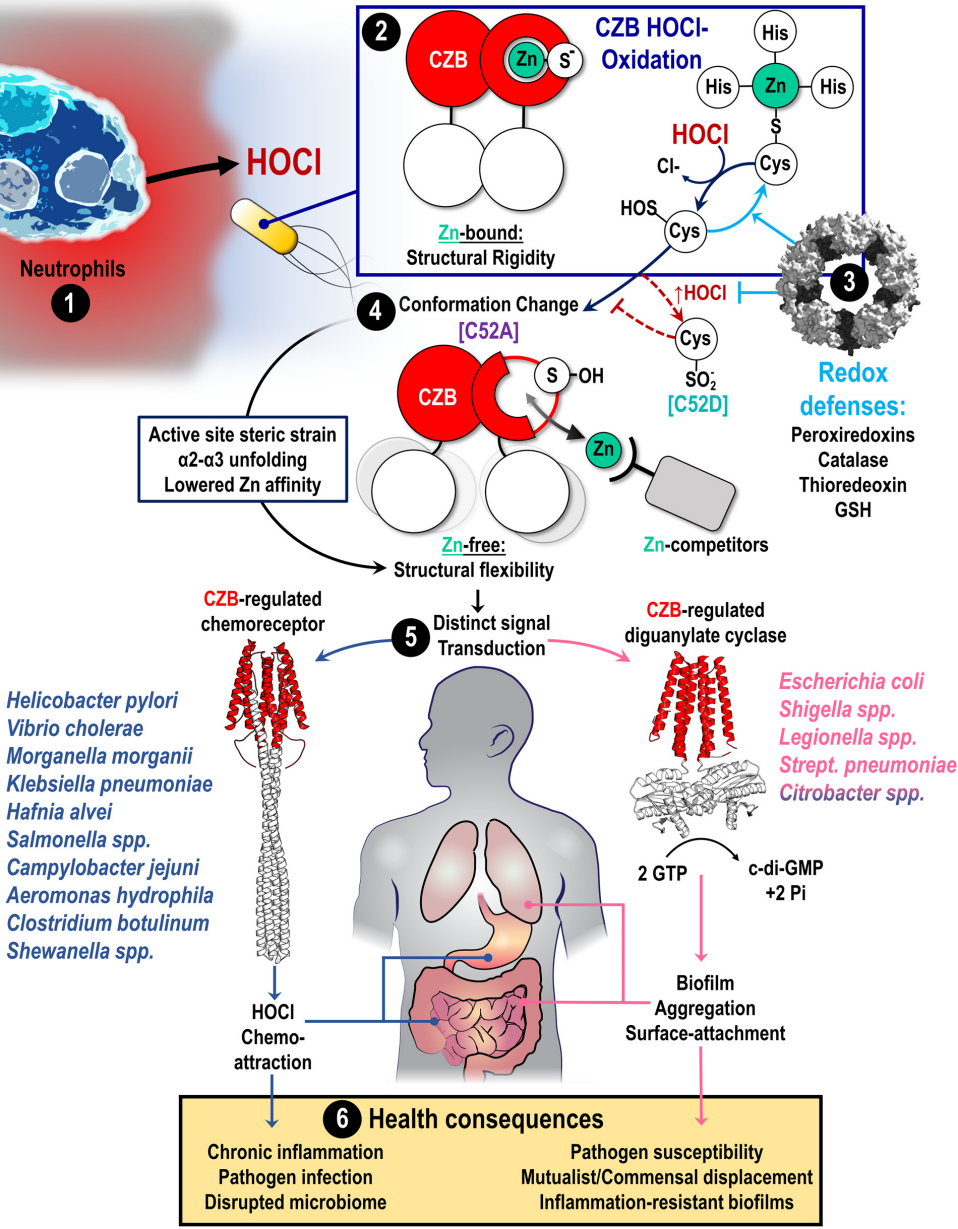
Proposed model for the role of CZB proteins in bacterial sensing of HOCl and implications for bacterial pathogenesis. (1) Neutrophils produce HOCl as part of innate immunity and inflammation to control bacterial populations and combat pathogens. (2) Bacterial CZB domains (red circles) exist as homodimers that bind zinc (green circle) in the low- to subfemtomolar range. Zinc binding elicits allosteric control of the full-length protein by promoting structural rigidity (white circles). CZB domains sense neutrophilic HOCl through the unique reactivity of their conserved zinc-thiolate complex and direct cysteine oxidation to form cysteine sulfenic acid (Cys-SOH). (3) CZB oxidation is reversed and inhibited by cellular reductants such as glutathione and, possibly, antioxidant enzymes. (4) The formation of Cys-SOH, modeled by the C52A mutant (violet), drives a large conformational change in the CZB domain through the active site strain induced by the Cys-SOH to promote local unfolding of the α2-α3 region, and this lowers the domain’s zinc-binding affinity. In the presence of cellular chelators that compete for zinc, this shifts the equilibrium toward the protein being zinc-free and promotes structural flexibility and increased dynamics. Alternatively, under conditions of high levels of HOCl (dark red dashed lines), the cysteine can react with a second molecule of HOCl to form cysteine sulfinate (Cys-SO_2_^−^), modeled by the C52D mutant (teal), which has higher zinc affinity and inhibits signal transduction. (5) Bacterial pathogens and pathobionts typically possess either CZB-regulated chemoreceptors (TlpD-like; blue) or CZB-regulated diguanylate cyclases (DgcZ-like; pink), and thus HOCl sensing is integrated into chemotaxis or c-di-GMP-signaling processes, respectively. The *Citrobacter* genus has some species with chemoreceptor forms and other species with diguanylate cyclase forms and is colored both blue and pink. (6) CZB sensing of HOCl may contribute to disease in a number of ways, such as initiating positive feedback loops that promote chronic inflammation through HOCl chemoattraction (e.g., H. pylori), stimulating inflammation to alter the tissue environment to the disadvantage of health-promoting native microbiota (e.g., S. enterica), or initiating virulence and inflammation resistance pathways in pathobiont communities (e.g., E. coli).

DgcZ is now the second CZB-containing protein found to be preferentially oxidized by HOCl over H_2_O_2_ (35). This result supports our earlier quantum mechanical study finding that chemoselectivity between these oxidants occurs as a result of geometric strain within the active site during an S_N_2-based reaction (52). An increasing number of bacterial proteins have been identified that, like CZB domains, appear to use a sulfur oxidation mechanism to respond selectively to HOCl. This observation supports that bacteria are attuned to the presence of this specific oxidant. It is interesting to speculate on why bacteria would discriminate between HOCl and other oxidants. One possibility is differences in cytotoxic potential. For example, H_2_O_2_ is also produced by neutrophils but is far less reactive and bactericidal than HOCl (76). Bacteria are also well equipped to eliminate hydroperoxides with enzymes such as peroxiredoxins (77, 78) and in some contexts even use H_2_O_2_ as a nutrient (79). But from the view of oxidants as molecular cues, how might bacteria interpret the presence of H_2_O_2_ versus HOCl? Bacteria can encounter H_2_O_2_ derived from many different sources, including immune responses, normal eukaryotic signaling (78), bacterial metabolism (80), dietary oxidants (81), and abiotic origins (82). Yet, with a few exceptions, the presence of hypohalous acids is emblematic of eukaryotic life. Myeloperoxidases are a major source of HOCl in animals (83), whereas vanadium peroxidases generate HOCl and HOBr in fungi and algae, respectively (84, 85). Thus, there is the potential that bacteria have evolved to interpret hypohalous acids as a signature of proximity to eukaryotes and multicellular organisms, and CZB domains may be a widespread mechanism by which bacteria integrate this information into decisions on localization. Based on our new mechanistic insights, we have developed a general model for how CZB domains facilitate HOCl sensing within animal hosts and the role this plays in colonization and disease, which we summarize in Fig. 7. Further work is required to test and substantiate this model.

### Signal transduction by CZB domains.

An important new insight into CZB function is that oxidation by HOCl regulates the domain’s affinity for Zn^2+^ and, hence, zinc-mediated allostery (Fig. 2 and 4). Specifically, the oxidation of the zinc-binding C52 to Cys-SOH facilitates the release of Zn^2+^ and results in the local unfolding of the α2-α3 region (Fig. 2, 3, and 7; see Fig. S2 in the supplemental material). Our modeling indicates that these conformational changes control the structural dynamics of the N (α1) and C (α5) termini that connect to regulated protein domains (Fig. 3C). These new insights enhance our understanding of CZB regulation of proteins, which can be understood as an order (zinc-bound)-to-disorder (zinc-free) transition. The structural basis by which zinc occupancy is transduced to the domain’s termini provides an explanation for how CZBs can use the same structural topology to regulate proteins like chemoreceptors, which mostly have C-terminal CZBs, and diguanylate cyclases, which have N-terminal CZBs (Fig. 6A). Presumably, the increased dynamics permit the population of conformations required for signal transduction. In the case of DgcZ-like proteins, this allows the GGDEF domain to align productively for c-di-GMP catalysis (34). For TlpD-like chemoreceptors, disorganization of the coiled-coil domain may serve to inhibit the autophosphorylation activity of the histidine kinase chemotaxis protein A (CheA) and promote chemoattraction (35).

We have observed an interesting pattern in which bacteria typically possess either chemoreceptor or diguanylate cyclase forms and not both (Fig. 6F). In the context of host-associated bacteria, this suggests that a conserved mechanism is utilized to sense host inflammation (the CZB domain) but that this signal is relayed into discrete lifestyle responses. The apparent incompatibility of these CZB protein forms could relate to the prerequisite of motility for chemotaxis. Differences in CZB regulatory outputs, i.e., bacterial attraction versus aggregation, may have important consequences for bacterial responses to inflammation and pathogenesis (Fig. 7).

### CZB domains can regulate the switching of biofilm lifestyle in response to HOCl.

We have shown in this work that the CZB-regulated diguanylate cyclase DgcZ acts as a direct sensor of exogenous HOCl to regulate c-di-GMP synthesis, surface attachment, and biofilm distribution. Our E. coli model biofilm system shows that DgcZ is required for increased biofilm formation in response to micromolar HOCl and that cell growth is not impaired under these conditions (Fig. 1). The conserved C52 of the CZB-binding core is required for biofilm responses to HOCl, and strains harboring C52 mutations that mimic cysteine oxidation display 2-fold-greater Congo red binding (Fig. 5A and B) and 1.5- to 2-fold-higher biofilm formation (Fig. 5C and D; Fig. S3A). The inability of the Δ*dgcZ* deletion strain and the catalytically inactivated *dgcZ^E208Q^* strain to recapitulate these responses suggests that these biofilm differences are due to direct regulation of DgcZ c-di-GMP production rather than indirect regulation of c-di-GMP signaling through effector proteins.

Interestingly, there were some differences between our *in vitro* biochemical analyses of the protein DgcZ and the cellular role of DgcZ in biofilm assays. First, only a modest increase in catalytic activity in response to low micromolar HOCl was observed for DgcZ protein *in vitro* (Fig. 4), whereas 1.5- to 6.2-fold increases in *dgcZ*-dependent biofilm occurred in response to HOCl treatments (Fig. 1 and 5). Such discrepancies between *in vitro* and *in vivo* responses are not unprecedented for diguanylate cyclases and can sometimes be attributed to feedbacks involving c-di-GMP effector proteins that are not present in the *in vitro* analyses (86). Alternatively, cellular reductants may alleviate nonspecific oxidation of DgcZ *in vivo* and allow activation responses at concentrations of HOCl higher than what we observed *in vitro*. Second, although we were able to use the C52A and C52D mutants as tools *in vitro* to model the Cys-SOH and Cys-SO_2_^−^ oxidation states, respectively, the biofilm formation of the *dgcZ^C52D^* strain was experiment dependent. *In vitro*, the C52D mutant protein had 10-fold-higher zinc affinity than the wild type, and its c-di-GMP catalysis was more readily inhibited by zinc (Fig. 2 and 4), but the *dgcZ^C52D^* strain behaved similarly to the *dgcZ^C52A^* strain in most assays and had increased Congo red binding and biofilm formation (Fig. 5; Fig. S3). Our *in vitro* studies on Cys-SOH (modeled by the C52A mutant) may be most relevant for understanding bacterial responses to physiological concentrations of HOCl, as bacteria are not known to use Cys-SO_2_^−^ (modeled by the C52D mutant) for signaling due to their inability to reduce and recover this overoxidized cysteine form (77).

### Roles for bacterial CZB sensing of HOCl in colonization and disease.

The relationship between inflammation and bacterial colonization of the human gastrointestinal tract is central to many diseases, as innate immune responses that fail to clear pathogens can manifest into states of chronic inflammation, causing tissue damage and even carcinogenesis (5, 11). In other cases, the inflammation induced by a bacterial pathogen can displace the native microbiota and promote dysbiosis, which is thought to contribute to the development of inflammatory bowel diseases (31, 32, 87–89). CZB domains and their function as HOCl sensors represent a new class of proteins that may play important roles in these processes. Our updated survey of CZB sequences provides the first quantification for what proteins and systems are regulated by CZB domains (Fig. 6; Data Set S1C). We expand on previous observations of the wide biological distribution of CZB domains (34) to quantitatively report that they are found in 21 bacterial phyla, as well as 6 candidate phyla, and are prevalent in *Proteobacteria*. These include many human gastrointestinal pathogens, such as species from the genera *Vibrio*, *Shewanella*, *Shigella*, *Helicobacter*, Campylobacter, *Citrobacter*, and Salmonella, and pathogens associated with nosocomial infections, such as *Legionella*, *Morganella*, Klebsiella, and Streptococcus (Fig. 6D and 7; Table 2; Data Set S1C).

Although much remains to be learned about the potential roles of CZB domains in bacterial infections, the c-di-GMP signaling processes we show to be regulated through CZB HOCl sensing are known to be involved in the virulence of E. coli. Adhesion of E. coli to intestinal microvilli is the primary mechanism of E. coli-induced diarrhea in humans (90), which kills over 50,000 individuals annually (91). An emerging body of evidence suggests that enduring and manipulating host inflammation is a central aspect of E. coli pathogenicity. CZB sensing of HOCl and stimulation of biofilm processes could play important roles in enabling the bacterium to overcome neutrophil responses and thrive in inflamed environments. For instance, Crohn’s disease colonoscopy biopsy specimens show dramatic increases in total E. coli (92). A majority of urinary tract infections are caused by uropathogenic E. coli (UPEC) (24), and a hallmark of the disease in patients is pyuria, stimulated through upregulation of the neutrophil chemokine interleukin-8 (50). Neutrophil infiltration of tissue has been proposed to benefit E. coli pathogenicity by stimulating adherence to epithelial cells (48), and DgcZ has been shown to facilitate adhesion to bladder cells (93). Our observations of the role of DgcZ in mediating biofilm distribution and pellicle formation in response to HOCl may provide new insights into the mechanisms of these diseases. Pellicle formation in E. coli is linked to the production of poly-GlcNAc (94), which our data suggest is robustly increased in response to HOCl, as modeled by the *dgcZ^C52A^* and *dgcZ^C52D^* strains (Fig. 5). A majority of clinical isolates of UPEC strains were found to promote virulence through production of poly-GlcNAc (95), and pellicle formation has also been found to be associated with enteropathogenic E. coli (EPEC) (68). Our findings on E. coli DgcZ may also be relevant for understanding chronic infections of the lungs, as S. pneumoniae and *Legionella* spp. possess DgcZ homologues (Fig. 7 and Table 2; Data Set S1C).

The function of CZB domains as mediators of chemotaxis and biofilm formation in response to inflammation may have roles in the organization and destabilization of host microbiomes. Bacterial populations of healthy human microbiomes that inhabit the low-oxygen environment of the gut are dominated by obligate anaerobic bacteria from the phyla *Bacteroidetes* and *Firmicutes*, with *Proteobacteria* species being less abundant (96, 97). However, the influx of neutrophils into the inflamed gastrointestinal tract stimulates a dramatic shift in the oxidant landscape (98) that can favor the opportunist. In diseases of chronic gut inflammation, such as colitis (99) and Crohn’s disease (100), the bacterial community structure changes substantially and a bloom in facultative aerobic *Gammaproteobacteria* is observed. It has been proposed that the expansion of *Gammaproteobacteria* species in these diseases is linked to their inherent ability to exploit host inflammation by utilizing biproducts of neutrophilic oxidants as nutrients (89). Tetrathionate and nitrate are two such metabolites that have been identified (28, 101–103). Therefore, HOCl could be a signal of opportunity sensed through CZB domains for bacteria able to tolerate and exploit inflamed tissue (Fig. 7). Intriguingly, it has been reported that the anti-inflammatory drug sulfasalazine, used for treatment of ulcerative colitis, is able to bind and directly inhibit DgcZ and E. coli biofilm formation (104), supporting the idea that CZB-regulated proteins may be therapeutic targets for interfering with bacterial coexistence with chronic inflammation.

### CZB indirect sensing of diverse effectors through zinc lability.

Having discussed the roles of CZB domains in sensing HOCl, we revisit previous observations of CZB-mediated responses to other stimuli. CZB-containing proteins have been implicated in cellular responses to superoxide (36–38), hydrogen peroxide (36), pH (39), Zn (34), Cu (40), Ni (40), Fe (36), and energy taxis (65), and no common mechanism has been proposed that could underlie these responses. While we did not directly test most of these effectors in our present work, our data do provide a possible explanation. The data in our study confirm that zinc binding is central to CZB function and allosteric control of proteins (34). This is apparent from our analysis of amino acid conservation among CZB-containing proteins in which CZB domains show strong conservation of only the regions containing, and proximal to, the zinc-binding core (Fig. 6B). This suggests that CZB responses to effectors are universally coordinated through the zinc-binding core. This does not exclude the possibility of effectors interacting with CZB domains through other means, such as protein-protein interactions, but these interactions are not conserved across distantly related bacteria.

A mechanism by which diverse stimuli could be perceived by CZB domains that requires only the zinc-binding core is through effector perturbation of cellular zinc homeostasis. For example, zinc solubility is heavily pH dependent (55), and cellular reductants like glutathione are abundant, readily bind zinc (105), and are responsive to the cellular redox state. What these shifts in zinc homeostasis indicate to bacteria, however, may be complex to interpret and system dependent. We speculate that CZB domains have two modes of sensing that rely both on zinc lability and the conserved zinc-binding core: (i) use of zinc as a cofactor for direct sensing of effectors that alter CZB zinc-binding affinity, such as HOCl, and (ii) use of zinc as a second messenger for indirect sensing of effectors that alter zinc homeostasis. Presumably, HOCl sensing is most relevant for bacteria that colonize animals and are exposed to millimolar concentrations of HOCl. However, as discussed above, sensing of hypohalous acids could serve functions in regulating interactions between bacteria and eukaryotes in nature. CZB sensing could be tuned through evolution to have broad or narrow spectrums of effector sensitivity. Our observation of distinct conservation patterns in the α3 region of functional classes of CZB proteins may be evidence of such evolutionary selection (Fig. 6C).

CZB homologs are present in diverse and evolutionarily distant bacteria, including enteric commensals and pathogens, soil-dwelling and marine species, and extremophiles such as the deep-sea genus *Thermatoga* (Fig. 6D). A corollary is that CZB domains have an ancient evolutionary heritage. Though difficulties exist in estimating bacterial protein origins due to horizontal gene transfer, a naive model based on a consensus of evolutionary divergence timelines indicates these proteins to have been present in the bacterial last universal common ancestor (LUCA) approximately four billion years ago (Fig. S4) (74). This suggests that the evolution of CZB domains predated eukaryotes, which originated approximately two billion years ago, and animals, which arose approximately 600 million years ago. Thus, the zinc-binding ability of CZB proteins can be viewed as its ancestral molecular function, and HOCl sensing is a more recent adaptation for interacting with eukaryotic life and colonizing animals.

## MATERIALS AND METHODS

### CZB conservation and phylogenetics.

Initial BLAST searches of the nonredundant protein database were performed to identify CZB-containing proteins using the CZB domains from H. pylori TlpD, E. coli DgcZ, and S. enterica McpA as search queries and the software Geneious Prime 2020 with default cutoffs. Automated identification of CZB domains was based on four attributes: (i) the presence of the conserved CZB zinc-binding 3His/1Cys core, (ii) the conserved CX(L/F)GXW(Y/L) motif identified in previous studies (33, 34), (iii) sequence coverage across the domain, and (iv) reasonable alignment to other confirmed CZB sequences. Sequences that did not meet these qualifications were reviewed manually. BLAST searches were continued iteratively with bona fide CZB sequences until no new sequences emerged. Protein sequences were annotated using Interpro (106) to identify protein domains and putative transmembrane regions. Sequences were aligned using MUSCLE (107), and relatedness trees were constructed with FastTree (108). Phylogenetic trees of CZB-containing organisms were constructed with phyloT (109), and divergence trees were constructed with TimeTree (74).

### Molecular dynamics simulation.

An intact model of the E. coli CZB homodimer (residues 7 to 126) was constructed based on PDB ID 3T9O (34). Coordinates for residues 38 to 51 from chain B were used to fill in the corresponding residues missing in chain A. Chemical alternations to residue 52 were accomplished using the psfgen program in VMD (110). Force-field parameter and topology files were obtained for cysteine sulfenic acid (Cys-SOH) from an independent study (111). All CZB models were hydrated with TIP3P and neutralized with 150 mM NaCl, producing systems containing ∼40,500 atoms. Each model was subjected to a conjugant-gradient energy minimization (2,000 steps), followed by a series of equilibration simulations with harmonic positional restraints applied successively to backbone+Zn (5 ns), Cα+Zn (5 ns), and Zn only (5 ns). Three independent, all-atom production simulations (1 μs each) provided the data used for subsequent analysis. All molecular dynamics simulations were performed using NAMD 2.13 (112) and the CHARMM36 force field (113). Simulations were conducted in the NPT ensemble (1 atm; 310 K) using a 2-fs timestep. Short-range, nonbonded interactions were calculated with a cutoff of 12 Å, and long-range electrostatics were evaluated using the particle mesh Ewald method. Molecular visualization and basic structural analyses were carried out in VMD. Representative videos of each simulation are presented in Movies S1A to S1E in the supplemental material.

### Quantum mechanical analyses.

All quantum mechanical analyses on the ligand exchange equilibria reported in this article were performed in Gaussian 16 (114). All structures were optimized and vibrational frequency calculations were performed in vacuum using B3LYP (115)/SDD (116) (Zn)/6-31+G (d,p) (117–119) level of theory (Data Set S1B). Electronic energies on the B3LYP-optimized geometries were calculated using the Minnesota functional M06 (120) suite and with the identical mixed basis sets described for optimizations. The optimized complexes were verified as ground states through vibrational frequency analysis (see the supplemental material for full authorship list for Gaussian 16). All thermal energies (e.g., Δ*G*) were calculated at 298 K and 1.0 atm. For each entry reported in Table 1, both sides of the equilibrium were modeled as the cationic Zn^2+^ species complexed with the appropriate exogenous ligand, that is, HOCl or H_2_O on the left side, and CH_3_S (H), CH_3_SO (H), or CH_3_SO_2_ (H) on the right side, depending on the oxidation state of the zinc-bound sulfur state being displaced.

### Recombinant protein purification.

For recombinant protein expression, Rosetta DE3 cells were transformed with pET28a plasmids from previous work (34) containing sequences for full-length DgcZ, DgcZ-C52A, and CZB (residues 1 to 128) containing 6×His affinity tags. Equivalent constructs of full-length DgcZ-C52D and CZB-C52D mutants were obtained commercially from GenScript as a service. Recombinant proteins were expressed and purified as described previously (34, 35). Frozen stocks were used to inoculate 25 ml of LB plus kanamycin (LB+Kan) (50 μg/ml) cultures and grown overnight. The following morning, 5 ml of these overnight cultures was added to each of four 1-liter cultures of LB+Kan and grown with shaking at 37°C until they reached an *A*_600_ of 0.6 to 0.8. Protein expression was induced with 1 mM IPTG (isopropyl-β-d-thiogalactopyranoside), grown for 3 h, and harvested by centrifugation. Cell pellets were resuspended into ice-cold lysis buffer containing 10 mM imidazole, 50 mM HEPES, 10% glycerol, 300 mM NaCl, and 0.5 mM TCEP [Tris(2-carboxyethyl)phosphine hydrochloride] (pH 7.9) and lysed by sonication. The cell suspension was centrifuged, and the soluble portion was retained for affinity chromatography. Lysate was applied to a prepacked gravity column of Ni-nitriloacetic acid (NTA) agarose beads (Qiagen) equilibrated with lysis buffer. Lysate was incubated with the beads for 10 min and then allowed to flow over the column twice. The column was then washed with lysis buffer until no protein was present in the flowthrough as determined using a Bradford assay. Purified protein was eluted by adding elution buffer to the column containing 300 mM imidazole, 50 mM HEPES, 300 mM NaCl, and 0.5 mM TCEP (pH 7.9), incubating the buffer in the column for 10 min, and then collecting the flowthrough in fractions. Samples were checked for purity by SDS-PAGE. Fractions containing pure protein were pooled and concentrated by use of a Pall centrifugal device with a 10-kDa cutoff and flash-frozen in liquid nitrogen. Prior to biochemical experiments, proteins were extensively dialyzed into buffers relevant for the reactions by using Thermo Scientific mini-dialysis tubes with 3- to 10-kDa molecular weight (MW) cutoffs.

### Cysteine-sulfenic acid quantification.

Reaction mixtures were prepared with purified protein (10 μM) dialyzed into PBS (pH 7), 500 μM 5,5-dimethyl-1,3-cyclohexanedione (dimedone), and additions of buffer or HOCl/H_2_O_2_ diluted into PBS buffer. The pHs of solutions prior to protein addition were monitored with a Toledo pH probe and adjusted as necessary with minimal additions of HCl. Reactions were allowed to proceed for 10 min at room temperature and then quenched with 100 μM l-methionine (Fig. 2B to D). For experiments with glutathione disulfide (GSSG), CZB protein was pretreated with 250 μM HOCl and subsequently with various GSSG treatments, followed by the addition of dimedone (Fig. 2E). GSSG was chosen to test the reversibility of CZB oxidation because it can form mixed disulfides with cysteines in both sulfenic acid and thiol states, and also is oxidized by HOCl, and therefore serves to fully quench the reaction. Cys-SOH formation was quantified by slot blotting analysis. Twenty-microliter volumes of samples were dispensed into 180 μl of quenching buffer containing 75 mM H_3_PO_4_–1 M NaCl and drawn by vacuum through a 0.4-μm polyvinylidene fluoride membrane in a 96-well slot blotter. The membrane was blocked in a buffer of 5% milk in 50 mM Tris, pH 7.5, 150 mM NaCl, 0.1% Tween 20 (TBST) for 10 min and incubated overnight at room temperature with rabbit anti-cysteine-dimedone antibody (Kerafast) at a 1:5,000 dilution. Subsequently the membrane was washed three times with 20 ml of TBST for a duration of 15 min and then incubated with goat anti-rabbit–horseradish peroxidase (HRP) secondary antibody (1:5,000) for 1 h. Afterward, the membrane was washed three times for 15 min with 20 ml of TBST and then visualized through chemiluminescence using an ECL kit and a Li-Cor imaging system.

### Fluorescence and circular dichroism assays.

The zinc-binding probe zinpyr-1 (Abcam) was used to detect available zinc through fluorescence emission. To avoid cross-reactivity of the probe with HOCl, samples were quenched with 1 mM methionine prior to probe addition, and probe was added no sooner than 10 min after quenching. Samples were analyzed in black, clear-bottom 96-well plates with excitation at 488 nm and emission spectra collected from 505 to 600 nm on a Spectramax i3 plate reader. Intrinsic protein fluorescence was collected similarly with excitation at 280 nm. Circular dichroism spectra were collected using a 1-mm quartz cuvette (Sterna Cells) on a Fluoromax-3 spectrofluorometer.

### DgcZ activity assays.

Reactions monitoring the *in vitro* production of c-di-GMP by DgcZ and cysteine point mutants were carried out in PBS buffer (10 mM Na_2_HPO_4_, 1.8 mM KH_2_PO_4_, 2.7 mM KCl, 137 mM NaCl, pH 7) with 5 mM MgCl_2_ and 300 or 500 μM GTP. Protein was preincubated in the reaction buffer for 10 min with HOCl, ZnCl_2_, dithiothreitol (DTT), or EDTA as necessary prior to the addition of GTP. Quantification of GTP and c-di-GMP at specific time points was performed as done previously (34) by chromatography using a Resource Q column and AKTA fast protein liquid chromatography (FPLC). A two-state model was fitted to the EDTA titration data with the amount of c-di-GMP produced being equal to *y* = *y*_min_ + Δ*y* and with Δ*y* given by the following quadratic equation:

$$\Delta y=0.5\times -\sqrt{\Delta y_{\text{max }}^{}{}^{2}\;-\;\left(2\;\Delta y_{\text{max }}\cdot L_{0}\right)\;+\;\left(2\;\Delta y_{\text{max }}\cdot K_{D}\right)\;+\;L_{0}^{}{}^{2}\;+\;\left(2L_{0}\cdot K_{D}\right)\;+\;\;K_{D}^{}{}^{2}}\;+\;\Delta y_{\text{max }}+\;L_{0}\;+\;K_{D}$$

where Δ*y*_max_ = *y*_max_ − *y*_min_ and *L*_0_ is the ligand concentration. Parameters to be refined were *y*_min_, *y*_max_, and the equilibrium dissociation constant, *K_D_*.

### Bacterial strains.

E. coli strains, genotypes, relevant phenotypes, and sources are listed in Table 3. All biofilm experiments were performed with MG1655-derivatized strains containing a *csrA* deletion (34, 42, 43).

**TABLE 3:** E. coli bacterial strains

Strain	Genotype	Relevant phenotype	Reference/source
AB958	*csrA*::Tn*5*Δ (kan)::Frt	*csrA* deletion, expresses wild-type *dgcZ* (*dgcZ^+^*)	Boehm et al., 2009 (43); Zähringer et al., 2013 (34)
AB959	*csrA*::Tn*5*Δ (kan)::(kan)::Frt Δ*ydeH*::Frt	Δ*dgcZ*, deletion	Zähringer et al., 2011, 2013 (34, 42)
AB1299	*csrA*::Tn*5*Δ (kan)::(kan)::Frt *ydeH* Frt *ydeH*-1 (E208Q)	*dgcZ^E208Q^*, catalytically inactive	Zähringer et al., 2011 (42)
AP1	*csrA*::Tn*5*Δ (kan)::Frt *ydeH* (C52A)	*dgcZ^C52A^* mutant	This work
AP2	*csrA*::Tn*5*Δ (kan)::Frt *ydeH* (C52D)	*dgcZ^C52D^* mutant	This work
Rosetta (DE3)	F^–^ *ompT hsdS_B_* (r_B_^–^ m_B_^–^) *gal dcm* (DE3) pRARE^2^ (Cam^r^)	Protein expression	Novagen

### Biofilm assays.

Unless specified otherwise, cells were prepared for biofilm assays with overnight growth with shaking in 5 ml of LB at 37°C, and fresh liquid LB cultures were inoculated in the morning and grown to the desired *A*_600_. Static biofilm assays with clear flat-bottom microplates were prepared with 200 μl of cells at an *A*_600_ of 0.5 or 1.0, covered with Parafilm, and grown for 16 or 24 h at 25 or 30°C as indicated. Static assays with 15-mm petri dishes were set up similarly but using 3 ml of cell culture at an *A*_600_ of 0.5, and holes were drilled into the lids to hold a 20-μl pipette with treatment solution. Treatments were applied by either direct addition, exposure to 20-μl treatment point sources with a Rainin 96-well liquidator, or exposure to a 20-μl Rainin pipette tip sealed with Parafilm containing 20 μl of treatment solution. Liquid culture rocking biofilm assays were conducted using 1 ml of cells at an *A*_600_ of 0.5 in LB medium in glass 10- by 75-mm culture tubes (Fisherbrand) and incubated upright at 30°C for 24 h with near-horizontal rocking.

Biofilm was quantified by crystal violet staining as done previously (34). Nonadhered cells were removed, and samples were washed twice with deionized water and stained with 0.1% crystal violet for 30 min. Excess stain was removed, and samples were washed twice with deionized water, dried, and then treated with a destain solution containing 30% methanol–10% acetic acid (equal in volume to cell culture) for 30 min. Samples were then quantified by measuring the absorbance at 562 nm. Quantifications of samples are presented as either raw *A*_562_ values or values normalized relative to the average of the wild-type untreated control strain in each experiment (*A*_562_ of sample/average *A*_562_ of untreated wild-type replicates). For quantification of biofilm distribution and pellicle formation, high-resolution microscopy images of crystal violet-stained culture tubes were captured on a Nikon Z20 dissecting scope and intensity profiles of tubes were collected along the tube length. Note that images of pellicle formation presented are illustrative and were not the images used for quantification. Quantification of pellicle formation was performed by integrating the intensity in the range of pixels 2000 to 2500.

### Congo red assays.

For assaying Congo red dye retention with growth on agar plates, 2 μl of cells either from overnight cultures or mid-log exponential growth cultures at an *A*_600_ of 0.5 was spotted onto LB agar plates containing 25 μg/ml Congo red dye and grown for 24 h at room temperature. Plates were imaged under identical lighting with a Leica MZ10F scope equipped with an MC190HD camera. Quantification of dye uptake was performed in ImageJ (121) with application of a red hue threshold of 1 to 14 in HSB color space. Images were then clustered into red, brown, and black (background) color bins using the Color Segmentation plugin and clustering pixels according to the k-means algorithm. Pixels from color-thresholded and clustered images were then counted using the Color Counter plugin. For visualizing Congo red staining of liquid cultures, cells were treated with 25 μg/ml dye and incubated for 10 min.

### Data availability.

Further information and requests for resources and reagents should be directed to and will be fulfilled by the lead contact, Arden Perkins (ardenp@uoregon.edu). Data values from figures and a curated database of CZB sequences are supplied in Data Set S1.

## References

[1] Hall-Stoodley L, Costerton JW, Stoodley P. 2004. Bacterial biofilms: from the natural environment to infectious diseases. Nat Rev Microbiol 2:95–108. doi:10.1038/nrmicro821. 15040259

[2] Schirmer TC. 2016. di-GMP synthesis: structural aspects of evolution, catalysis and regulation. J Mol Biol 428:3683–3701. doi:10.1016/j.jmb.2016.07.023. 27498163

[3] Wadhams GH, Armitage JP. 2004. Making sense of it all: bacterial chemotaxis. Nat Rev Mol Cell Biol 5:1024–1037. doi:10.1038/nrm1524. 15573139

[4] Hazelbauer GL, Falke JJ, Parkinson JS. 2008. Bacterial chemoreceptors: high-performance signaling in networked arrays. Trends Biochem Sci 33:9–19. doi:10.1016/j.tibs.2007.09.014. 18165013 PMC2890293

[5] Sultana S, Foti A, Dahl J-U. 2020. Bacterial defense systems against the neutrophilic oxidant hypochlorous acid. Infect Immun 88:e00964-19. doi:10.1128/IAI.00964-19. 32152198 PMC7309615

[6] Klebanoff SJ, Kettle AJ, Rosen H, Winterbourn CC, Nauseef WM. 2013. Myeloperoxidase: a front-line defender against phagocytosed microorganisms. J Leukoc Biol 93:185–198. doi:10.1189/jlb.0712349. 23066164 PMC3545676

[7] Degrossoli A, Müller A, Xie K, Schneider JF, Bader V, Winklhofer KF, Meyer AJ, Leichert LI. 2018. Neutrophil-generated HOCl leads to non-specific thiol oxidation in phagocytized bacteria. Elife 7:e32288. doi:10.7554/eLife.32288. 29506649 PMC5839695

[8] Loughran NB, Hinde S, McCormick-Hill S, Leidal KG, Bloomberg S, Loughran ST, O'Connor B, O'Fágáin C, Nauseef WM, O'Connell MJ. 2012. Functional consequence of positive selection revealed through rational mutagenesis of human myeloperoxidase. Mol Biol Evol 29:2039–2046. doi:10.1093/molbev/mss073. 22355012 PMC3408071

[9] Palmer LJ, Cooper PR, Ling MR, Wright HJ, Huissoon A, Chapple ILC. 2012. Hypochlorous acid regulates neutrophil extracellular trap release in humans. Clin Exp Immunol 167:261–268. doi:10.1111/j.1365-2249.2011.04518.x. 22236002 PMC3278692

[10] Halliwell B, Gutteridge JMC. 2015. Free radicals in biology and medicine. Oxford University Press, Oxford, United Kingdom.

[11] Test ST, Weiss SJ. 1986. The generation of utilization of chlorinated oxidants by human neutrophils. Adv Free Radic Biol Med 2:91–116. doi:10.1016/S8755-9668(86)80025-4.

[12] Winterbourn CC, Hampton MB, Livesey JH, Kettle AJ. 2006. Modeling the reactions of superoxide and myeloperoxidase in the neutrophil phagosome: implications for microbial killing. J Biol Chem 281:39860–39869. doi:10.1074/jbc.M605898200. 17074761

[13] Lebrun V, Ravanat J-L, Latour J-M, Sénèque O. 2016. Near diffusion-controlled reaction of a Zn(Cys) 4 zinc finger with hypochlorous acid. Chem Sci 7:5508–5516. doi:10.1039/c6sc00974c. 30034691 PMC6021785

[14] Cook NL, Pattison DI, Davies MJ. 2012. Myeloperoxidase-derived oxidants rapidly oxidize and disrupt zinc-cysteine/histidine clusters in proteins. Free Radic Biol Med 53:2072–2080. doi:10.1016/j.freeradbiomed.2012.09.033. 23032100

[15] Fliss H, Ménard M, Desai M. 1991. Hypochlorous acid mobilizes cellular zinc. Can J Physiol Pharmacol 69:1686–1691. doi:10.1139/y91-250. 1666536

[16] Tatsumi T, Fliss H. 1994. Hypochlorous acid and chloramines increase endothelial permeability: possible involvement of cellular zinc. Am J Physiol 267:H1597–H1607. doi:10.1152/ajpheart.1994.267.4.H1597. 7943407

[17] Gray MJ, Wholey W-Y, Parker BW, Kim M, Jakob U. 2013. NemR is a bleach-sensing transcription factor. J Biol Chem 288:13789–13798. doi:10.1074/jbc.M113.454421. 23536188 PMC3650415

[18] Wan F, Yin J, Sun W, Gao H. 2019. Oxidized OxyR up-regulates ahpCF expression to suppress plating defects of oxyR- and catalase-deficient strains. Front Microbiol 10:439. doi:10.3389/fmicb.2019.00439. 30899252 PMC6416212

[19] Gundlach J, Winter J. 2014. Evolution of Escherichia coli for maximum HOCl resistance through constitutive expression of the OxyR regulon. Microbiology (Reading) 160:1690–1704. doi:10.1099/mic.0.074815-0. 24899627

[20] Drazic A, Miura H, Peschek J, Le Y, Bach NC, Kriehuber T, Winter J. 2013. Methionine oxidation activates a transcription factor in response to oxidative stress. Proc Natl Acad Sci U S A 110:9493–9498. doi:10.1073/pnas.1300578110. 23690622 PMC3677443

[21] Parker BW, Schwessinger EA, Jakob U, Gray MJ. 2013. The RclR protein is a reactive chlorine-specific transcription factor in Escherichia coli. J Biol Chem 288:32574–32584. doi:10.1074/jbc.M113.503516. 24078635 PMC3820890

[22] Winter J, Ilbert M, Graf PCF, Özcelik D, Jakob U. 2008. Bleach activates a redox-regulated chaperone by oxidative protein unfolding. Cell 135:691–701. doi:10.1016/j.cell.2008.09.024. 19013278 PMC2606091

[23] Goemans CV, Vertommen D, Agrebi R, Collet J-F. 2018. CnoX is a chaperedoxin: a holdase that protects its substrates from irreversible oxidation. Mol Cell 70:614–627.e7. doi:10.1016/j.molcel.2018.04.002. 29754824

[24] Terlizzi ME, Gribaudo G, Maffei ME. 2017. Uropathogenic Escherichia coli (UPEC) infections: virulence factors, bladder responses, antibiotic, and non-antibiotic antimicrobial strategies. Front Microbiol 8:1566. doi:10.3389/fmicb.2017.01566. 28861072 PMC5559502

[25] Abdel-Nour M, Duncan C, Low DE, Guyard C. 2013. Biofilms: the stronghold of Legionella pneumophila. Int J Mol Sci 14:21660–21675. doi:10.3390/ijms141121660. 24185913 PMC3856027

[26] Newton HJ, Ang DKY, van Driel IR, Hartland EL. 2010. Molecular pathogenesis of infections caused by Legionella pneumophila. Clin Microbiol Rev 23:274–298. doi:10.1128/CMR.00052-09. 20375353 PMC2863363

[27] Marks LR, Reddinger RM, Hakansson AP. 2014. Biofilm formation enhances fomite survival of Streptococcus pneumoniae and Streptococcus pyogenes. Infect Immun 82:1141–1146. doi:10.1128/IAI.01310-13. 24371220 PMC3957990

[28] Rivera-Chávez F, Winter SE, Lopez CA, Xavier MN, Winter MG, Nuccio SP, Russell JM, Laughlin RC, Lawhon SD, Sterzenbach T, Bevins CL, Tsolis RM, Harshey R, Adams LG, Bäumler AJ. 2013. Salmonella uses energy taxis to benefit from intestinal inflammation. PLoS Pathog 9:e1003267. doi:10.1371/journal.ppat.1003267. 23637594 PMC3630101

[29] Aihara E, Closson C, Matthis AL, Schumacher MA, Engevik AC, Zavros Y, Ottemann KM, Montrose MH. 2014. Motility and chemotaxis mediate the preferential colonization of gastric injury sites by Helicobacter pylori. PLoS Pathog 10:e1004275. doi:10.1371/journal.ppat.1004275. 25033386 PMC4102597

[30] Rolig AS, Shanks J, Carter JE, Ottemann KM. 2012. Helicobacter pylori requires TlpD-driven chemotaxis to proliferate in the antrum. Infect Immun 80:3713–3720. doi:10.1128/IAI.00407-12. 22802346 PMC3457577

[31] Schirmer M, Garner A, Vlamakis H, Xavier RJ. 2019. Microbial genes and pathways in inflammatory bowel disease. Nat Rev Microbiol 17:497–511. doi:10.1038/s41579-019-0213-6. 31249397 PMC6759048

[32] Plichta DR, Graham DB, Subramanian S, Xavier RJ. 2019. Therapeutic opportunities in inflammatory bowel disease: mechanistic dissection of host-microbiome relationships. Cell 178:1041–1056. doi:10.1016/j.cell.2019.07.045. 31442399 PMC6778965

[33] Draper J, Karplus K, Ottemann KM. 2011. Identification of a chemoreceptor zinc-binding domain common to cytoplasmic bacterial chemoreceptors. J Bacteriol 193:4338–4345. doi:10.1128/JB.05140-11. 21725005 PMC3165512

[34] Zähringer F, Lacanna E, Jenal U, Schirmer T, Boehm A. 2013. Structure and signaling mechanism of a zinc-sensory diguanylate cyclase. Structure 21:1149–1157. doi:10.1016/j.str.2013.04.026. 23769666

[35] Perkins A, Tudorica DA, Amieva MR, Remington SJ, Guillemin K. 2019. Helicobacter pylori senses bleach (HOCl) as a chemoattractant using a cytosolic chemoreceptor. PLoS Biol 17:e3000395. doi:10.1371/journal.pbio.3000395. 31465435 PMC6715182

[36] Collins KD, Andermann TM, Draper J, Sanders L, Williams SM, Araghi C, Ottemann KM. 2016. The Helicobacter pylori CZB cytoplasmic chemoreceptor TlpD forms an autonomous polar chemotaxis signaling complex that mediates a tactic response to oxidative stress. J Bacteriol 198:1563–1575. doi:10.1128/JB.00071-16. 27002127 PMC4959281

[37] Behrens W, Schweinitzer T, McMurry JL, Loewen PC, Buettner FFR, Menz S, Josenhans C. 2016. Localisation and protein-protein interactions of the Helicobacter pylori taxis sensor TlpD and their connection to metabolic functions. Sci Rep 6:23582. doi:10.1038/srep23582. 27045738 PMC4820699

[38] Lacanna E, Bigosch C, Kaever V, Boehm A, Becker A. 2016. Evidence for Escherichia coli diguanylate cyclase DgcZ interlinking surface sensing and adhesion via multiple regulatory routes. J Bacteriol 198:2524–2535. doi:10.1128/JB.00320-16. 27402625 PMC4999941

[39] Huang JY, Sweeney EG, Guillemin K, Amieva MR. 2017. Multiple acid sensors control Helicobacter pylori colonization of the stomach. PLoS Pathog 13:e1006118. doi:10.1371/journal.ppat.1006118. 28103315 PMC5245789

[40] Sanders L, Andermann TM, Ottemann KM. 2013. A supplemented soft agar chemotaxis assay demonstrates the Helicobacter pylori chemotactic response to zinc and nickel. Microbiology 159:46–57. doi:10.1099/mic.0.062877-0. 23139399 PMC3542728

[41] Fung C, Tan S, Nakajima M, Skoog EC, Camarillo-Guerrero LF, Klein JA, Lawley TD, Solnick JV, Fukami T, Amieva MR. 2019. High-resolution mapping reveals that microniches in the gastric glands control Helicobacter pylori colonization of the stomach. PLoS Biol 17:e3000231. doi:10.1371/journal.pbio.3000231. 31048876 PMC6497225

[42] Zähringer F, Massa C, Schirmer T. 2011. Efficient enzymatic production of the bacterial second messenger c-di-GMP by the diguanylate cyclase YdeH from E. coli. Appl Biochem Biotechnol 163:71–79. doi:10.1007/s12010-010-9017-x. 20582742

[43] Boehm A, Steiner S, Zaehringer F, Casanova A, Hamburger F, Ritz D, Keck W, Ackermann M, Schirmer T, Jenal U. 2009. Second messenger signalling governs Escherichia coli biofilm induction upon ribosomal stress. Mol Microbiol 72:1500–1516. doi:10.1111/j.1365-2958.2009.06739.x. 19460094

[44] Boehm A, Kaiser M, Li H, Spangler C, Kasper CA, Ackermann M, Kaever V, Sourjik V, Roth V, Jenal U. 2010. Second messenger-mediated adjustment of bacterial swimming velocity. Cell 141:107–116. doi:10.1016/j.cell.2010.01.018. 20303158

[45] Hengge R. 2009. Principles of c-di-GMP signalling in bacteria. Nat Rev Microbiol 7:263–273. doi:10.1038/nrmicro2109. 19287449

[46] Vestby LK, Grønseth T, Simm R, Nesse LL. 2020. Bacterial biofilm and its role in the pathogenesis of disease. Antibiotics (Basel) 9:59. doi:10.3390/antibiotics9020059. PMC7167820 32028684

[47] DePas WH, Hufnagel DA, Lee JS, Blanco LP, Bernstein HC, Fisher ST, James GA, Stewart PS, Chapman MR. 2013. Iron induces bimodal population development by Escherichia coli. Proc Natl Acad Sci U S A 110:2629–2634. doi:10.1073/pnas.1218703110. 23359678 PMC3574911

[48] Boll EJ, Struve C, Sander A, Demma Z, Krogfelt KA, McCormick BA. 2012. Enteroaggregative Escherichia coli promotes transepithelial migration of neutrophils through a conserved 12-lipoxygenase pathway. Cell Microbiol 14:120–132. doi:10.1111/j.1462-5822.2011.01706.x. 21951973 PMC4089036

[49] Michail SK, Halm DR, Abernathy F. 2003. Enteropathogenic Escherichia coli: stimulating neutrophil migration across a cultured intestinal epithelium without altering transepithelial conductance. J Pediatr Gastroenterol Nutr 36:253–260. doi:10.1097/00005176-200302000-00018. 12548063

[50] Mulvey MA, Schilling JD, Martinez JJ, Hultgren SJ. 2000. Bad bugs and beleaguered bladders: interplay between uropathogenic Escherichia coli and innate host defenses. Proc Natl Acad Sci U S A 97:8829–8835. doi:10.1073/pnas.97.16.8829. 10922042 PMC34019

[51] Jonas K, Edwards AN, Simm R, Romeo T, Römling U, Melefors O. 2008. The RNA binding protein CsrA controls cyclic di-GMP metabolism by directly regulating the expression of GGDEF proteins. Mol Microbiol 70:236–257. doi:10.1111/j.1365-2958.2008.06411.x. 18713317 PMC2735045

[52] Zumwalt L, Perkins A, Ogba OM. 2020. Mechanism and chemoselectivity for HOCl-mediated oxidation of zinc-bound thiolates. Chemphyschem 21:2384–2387. doi:10.1002/cphc.202000634. doi:10.1002/cphc.202000634. 32915482

[53] Seo YH, Carroll KS. 2009. Profiling protein thiol oxidation in tumor cells using sulfenic acid-specific antibodies. Proc Natl Acad Sci U S A 106:16163–16168. doi:10.1073/pnas.0903015106. 19805274 PMC2741475

[54] Colvin RA, Holmes WR, Fontaine CP, Maret W. 2010. Cytosolic zinc buffering and muffling: their role in intracellular zinc homeostasis. Metallomics 2:306–317. doi:10.1039/b926662c. 21069178

[55] Krężel A, Maret W. 2016. The biological inorganic chemistry of zinc ions. Arch Biochem Biophys 611:3–19. doi:10.1016/j.abb.2016.04.010. 27117234 PMC5120989

[56] Outten CE, O’Halloran TV. 2001. Femtomolar sensitivity of metalloregulatory proteins controlling zinc homeostasis. Science 292:2488–2492. doi:10.1126/science.1060331. 11397910

[57] Gilston BA, Wang S, Marcus MD, Canalizo-Hernández MA, Swindell EP, Xue Y, Mondragón A, O'Halloran TV. 2014. Structural and mechanistic basis of zinc regulation across the E. coli Zur regulon. PLoS Biol 12:e1001987. doi:10.1371/journal.pbio.1001987. 25369000 PMC4219657

[58] Lapenna D, De Gioia S, Ciofani G, Mezzetti A, Consoli A, Di Ilio C, Cuccurullo F. 1994. Hypochlorous acid-induced zinc release from thiolate bonds: a potential protective mechanism towards biomolecules oxidant damage during inflammation. Free Radic Res 20:165–170. doi:10.3109/10715769409147513. 7912612

[59] Woodroofe CC, Masalha R, Barnes KR, Frederickson CJ, Lippard SJ. 2004. Membrane-permeable and -impermeable sensors of the Zinpyr family and their application to imaging of hippocampal zinc in vivo. Chem Biol 11:1659–1666. doi:10.1016/j.chembiol.2004.09.013. 15610850

[60] Kabsch W, Sander C. 1983. Dictionary of protein secondary structure: pattern recognition of hydrogen-bonded and geometrical features. Biopolymers 22:2577–2637. doi:10.1002/bip.360221211. 6667333

[61] Roos G, Foloppe N, Messens J. 2012. Understanding the pKa of redox cysteines: the key role of hydrogen bonding. Antioxid Redox Signal 18:94–127. doi:10.1089/ars.2012.4521. 22746677

[62] Claiborne A, Miller H, Parsonage D, Ross RP. 1993. Protein-sulfenic acid stabilization and function in enzyme catalysis and gene regulation. FASEB J 7:1483–1490. doi:10.1096/fasebj.7.15.8262333. 8262333

[63] McGrath AJ, Garrett GE, Valgimigli L, Pratt DA. 2010. The redox chemistry of sulfenic acids. J Am Chem Soc 132:16759–16761. doi:10.1021/ja1083046. 21049943

[64] Burkhard RK, Sellers DE, DeCou F, Lambert JL. 1959. The pKa’s of aromatic sulfinic acids. J Org Chem 24:767–769. doi:10.1021/jo01088a010.

[65] Behrens W, Schweinitzer T, Bal J, Dorsch M, Bleich A, Kops F, Brenneke B, Didelot X, Suerbaum S, Josenhans C. 2013. Role of energy sensor TlpD of Helicobacter pylori in gerbil colonization and genome analyses after adaptation in the gerbil. Infect Immun 81:3534–3551. doi:10.1128/IAI.00750-13. 23836820 PMC3811781

[66] Gupta V, Carroll KS. 2014. Sulfenic acid chemistry, detection and cellular lifetime. Biochim Biophys Acta 1840:847–875. doi:10.1016/j.bbagen.2013.05.040. 23748139 PMC4184475

[67] Perkins A, Parsonage D, Nelson KJ, Ogba OM, Cheong PH-Y, Poole LB, Karplus PA. 2016. Peroxiredoxin catalysis at atomic resolution. Structure 24:1668–1678. doi:10.1016/j.str.2016.07.012. 27594682 PMC5241139

[68] Weiss-Muszkat M, Shakh D, Zhou Y, Pinto R, Belausov E, Chapman MR, Sela S. 2010. Biofilm formation by and multicellular behavior of Escherichia coli O55:H7, an atypical enteropathogenic strain. Appl Environ Microbiol 76:1545–1554. doi:10.1128/AEM.01395-09. 20080991 PMC2832381

[69] Reichhardt C, Jacobson AN, Maher MC, Uang J, McCrate OA, Eckart M, Cegelski L. 2015. Congo Red interactions with curli-producing E. coli and native curli amyloid fibers. PLoS One 10:e0140388. doi:10.1371/journal.pone.0140388. 26485271 PMC4618944

[70] Johnson M, Zaretskaya I, Raytselis Y, Merezhuk Y, McGinnis S, Madden TL. 2008. NCBI BLAST: a better web interface. Nucleic Acids Res 36:W5–W9. doi:10.1093/nar/gkn201. 18440982 PMC2447716

[71] Tubiana J, Cocco S, Monasson R. 2019. Learning protein constitutive motifs from sequence data. Elife 8:e39397. doi:10.7554/eLife.39397. 30857591 PMC6436896

[72] Perkins A, Gretes MC, Nelson KJ, Poole LB, Karplus PA. 2012. Mapping the active site helix-to-strand conversion of CxxxxC peroxiredoxin Q enzymes. Biochemistry 51:7638–7650. doi:10.1021/bi301017s. 22928725 PMC3549014

[73] Sweeney EG, Perkins A, Kallio K, James Remington S, Guillemin K. 2018. Structures of the ligand-binding domain of Helicobacter pylori chemoreceptor TlpA. Protein Sci 27:1961–1968. doi:10.1002/pro.3503. 30171638 PMC6201720

[74] Kumar S, Stecher G, Suleski M, Hedges SB. 2017. TimeTree: a resource for timelines, timetrees, and divergence times. Mol Biol Evol 34:1812–1819. doi:10.1093/molbev/msx116. 28387841

[75] Collins KD, Lacal J, Ottemann KM. 2014. Internal sense of direction: sensing and signaling from cytoplasmic chemoreceptors. Microbiol Mol Biol Rev 78:672–684. doi:10.1128/MMBR.00033-14. 25428939 PMC4248653

[76] Bonvillain RW, Painter RG, Ledet EM, Wang G. 2011. Comparisons of resistance of CF and non-CF pathogens to hydrogen peroxide and hypochlorous acid oxidants in vitro. BMC Microbiol 11:112. doi:10.1186/1471-2180-11-112. 21599970 PMC3118099

[77] Perkins A, Poole LB, Karplus PA. 2014. Tuning of peroxiredoxin catalysis for various physiological roles. Biochemistry 53:7693–7705. doi:10.1021/bi5013222. 25403613 PMC4270387

[78] Perkins A, Nelson KJ, Parsonage D, Poole LB, Karplus PA. 2015. Peroxiredoxins: guardians against oxidative stress and modulators of peroxide signaling. Trends Biochem Sci 40:435–445. doi:10.1016/j.tibs.2015.05.001. 26067716 PMC4509974

[79] Miller BM, Liou MJ, Zhang LF, Nguyen H, Litvak Y, Schorr EM, Jang KK, Tiffany CR, Butler BP, Bäumler AJ. 2020. Anaerobic respiration of NOX1-derived hydrogen peroxide licenses bacterial growth at the colonic surface. Cell Host Microbe 28:789–797.e5. doi:10.1016/j.chom.2020.10.009. 33301718 PMC7758056

[80] Hertzberger R, Arents J, Dekker HL, Pridmore RD, Gysler C, Kleerebezem M, de Mattos MJ. 2014. H_2_O_2_ production in species of the Lactobacillus acidophilus group: a central role for a novel NADH-dependent flavin reductase. Appl Environ Microbiol 80:2229–2239. doi:10.1128/AEM.04272-13. 24487531 PMC3993133

[81] Halliwell B, Clement MV, Long LH. 2000. Hydrogen peroxide in the human body. FEBS Lett 486:10–13. doi:10.1016/S0014-5793(00)02197-9. 11108833

[82] Cooper WJ, Zika RG. 1983. Photochemical formation of hydrogen peroxide in surface and ground waters exposed to sunlight. Science 220:711–712. doi:10.1126/science.220.4598.711. 17813875

[83] Daiyasu H, Toh H. 2000. Molecular evolution of the myeloperoxidase family. J Mol Evol 51:433–445. doi:10.1007/s002390010106. 11080366

[84] Butler A, N Carter-Franklin J. 2004. The role of vanadium bromoperoxidase in the biosynthesis of halogenated marine natural products. Nat Prod Rep 21:180–188. doi:10.1039/b302337k. 15039842

[85] Winter JM, Moore BS. 2009. Exploring the chemistry and biology of vanadium-dependent haloperoxidases. J Biol Chem 284:18577–18581. doi:10.1074/jbc.R109.001602. 19363038 PMC2707250

[86] Giacalone D, Smith TJ, Collins AJ, Sondermann H, Koziol LJ, O’Toole GA. 2018. Ligand-mediated biofilm formation via enhanced physical interaction between a diguanylate cyclase and its receptor. mBio 9:e01254-18. doi:10.1128/mBio.01254-18. 29991582 PMC6050961

[87] Gradel KO, Nielsen HL, Schønheyder HC, Ejlertsen T, Kristensen B, Nielsen H. 2009. Increased short- and long-term risk of inflammatory bowel disease after Salmonella or Campylobacter gastroenteritis. Gastroenterology 137:495–501. doi:10.1053/j.gastro.2009.04.001. 19361507

[88] Stecher B, Robbiani R, Walker AW, Westendorf AM, Barthel M, Kremer M, Chaffron S, Macpherson AJ, Buer J, Parkhill J, Dougan G, von Mering C, Hardt W-D. 2007. Salmonella enterica serovar Typhimurium exploits inflammation to compete with the intestinal microbiota. PLoS Biol 5:e244. doi:10.1371/journal.pbio.0050244. PMC1951780 17760501

[89] Winter SE, Bäumler AJ. 2014. Dysbiosis in the inflamed intestine. Gut Microbes 5:71–73. doi:10.4161/gmic.27129. 24637596 PMC4049941

[90] Schuller S, Lucas M, Kaper JB, Girón JA, Phillips AD. 2009. The ex vivo response of human intestinal mucosa to enteropathogenic Escherichia coli infection. Cell Microbiol 11:521–530. doi:10.1111/j.1462-5822.2008.01275.x. 19134113 PMC2676445

[91] Khalil IA, Troeger C, Blacker BF, Rao PC, Brown A, Atherly DE, Brewer TG, Engmann CM, Houpt ER, Kang G, Kotloff KL, Levine MM, Luby SP, MacLennan CA, Pan WK, Pavlinac PB, Platts-Mills JA, Qadri F, Riddle MS, Ryan ET, Shoultz DA, Steele AD, Walson JL, Sanders JW, Mokdad AH, Murray CJL, Hay SI, Reiner RC, Jr. 2018. Morbidity and mortality due to shigella and enterotoxigenic Escherichia coli diarrhoea: the Global Burden of Disease Study 1990–2016. Lancet Infect Dis 18:1229–1240. doi:10.1016/S1473-3099(18)30475-4. 30266330 PMC6202441

[92] Rhodes JM. 2007. The role of Escherichia coli in inflammatory bowel disease. Gut 56:610–612. doi:10.1136/gut.2006.111872. 17440180 PMC1942130

[93] Spurbeck RR, Tarrien RJ, Mobley HLT. 2012. Enzymatically active and inactive phosphodiesterases and diguanylate cyclases are involved in regulation of motility or sessility in Escherichia coli CFT073. mBio 3:e00307-12. doi:10.1128/mBio.00307-12. 23047748 PMC3484386

[94] Wang X, Preston JF, Romeo T. 2004. The pgaABCD locus of Escherichia coli promotes the synthesis of a polysaccharide adhesin required for biofilm formation. J Bacteriol 186:2724–2734. doi:10.1128/JB.186.9.2724-2734.2004. 15090514 PMC387819

[95] Cerca N, Maira-Litrán T, Jefferson KK, Grout M, Goldmann DA, Pier GB. 2007. Protection against Escherichia coli infection by antibody to the Staphylococcus aureus poly-N-acetylglucosamine surface polysaccharide. Proc Natl Acad Sci U S A 104:7528–7533. doi:10.1073/pnas.0700630104. 17446272 PMC1863476

[96] Eckburg PB, Bik EM, Bernstein CN, Purdom E, Dethlefsen L, Sargent M, Gill SR, Nelson KE, Relman DA. 2005. Diversity of the human intestinal microbial flora. Science 308:1635–1638. doi:10.1126/science.1110591. 15831718 PMC1395357

[97] Lloyd-Price J, Arze C, Ananthakrishnan AN, Schirmer M, Avila-Pacheco J, Poon TW, Andrews E, Ajami NJ, Bonham KS, Brislawn CJ, Casero D, Courtney H, Gonzalez A, Graeber TG, Hall AB, Lake K, Landers CJ, Mallick H, Plichta DR, Prasad M, Rahnavard G, Sauk J, Shungin D, Vázquez-Baeza Y, White RA III, IBDMDB Investigators, Braun J, Denson LA, Jansson JK, Knight R, Kugathasan S, McGovern DPB, Petrosino JF, Stappenbeck TS, Winter HS, Clish CB, Franzosa EA, Vlamakis H, Xavier RJ, Huttenhower C. 2019. Multi-omics of the gut microbial ecosystem in inflammatory bowel diseases. Nature 569:655–662. doi:10.1038/s41586-019-1237-9. 31142855 PMC6650278

[98] Wei P, Yuan W, Xue F, Zhou W, Li R, Zhang D, Yi T. 2018. Deformylation reaction-based probe for in vivo imaging of HOCl. Chem Sci 9:495–501. doi:10.1039/c7sc03784h. 29619205 PMC5868080

[99] Lupp C, Robertson ML, Wickham ME, Sekirov I, Champion OL, Gaynor EC, Finlay BB. 2007. Host-mediated inflammation disrupts the intestinal microbiota and promotes the overgrowth of Enterobacteriaceae. Cell Host Microbe 2:204. doi:10.1016/j.chom.2007.08.002. 18030708

[100] Frank DN, St Amand AL, Feldman RA, Boedeker EC, Harpaz N, Pace NR. 2007. Molecular-phylogenetic characterization of microbial community imbalances in human inflammatory bowel diseases. Proc Natl Acad Sci U S A 104:13780–13785. doi:10.1073/pnas.0706625104. 17699621 PMC1959459

[101] Winter SE, Winter MG, Xavier MN, Thiennimitr P, Poon V, Keestra AM, Laughlin RC, Gomez G, Wu J, Lawhon SD, Popova IE, Parikh SJ, Adams LG, Tsolis RM, Stewart VJ, Bäumler AJ. 2013. Host-derived nitrate boosts growth of E. coli in the inflamed gut. Science 339:708–711. doi:10.1126/science.1232467. 23393266 PMC4004111

[102] Winter SE, Thiennimitr P, Winter MG, Butler BP, Huseby DL, Crawford RW, Russell JM, Bevins CL, Adams LG, Tsolis RM, Roth JR, Bäumler AJ. 2010. Gut inflammation provides a respiratory electron acceptor for *Salmonella*. Nature 467:426–429. doi:10.1038/nature09415. 20864996 PMC2946174

[103] Spees AM, Wangdi T, Lopez CA, Kingsbury DD, Xavier MN, Winter SE, Tsolis RM, Bäumler AJ. 2013. Streptomycin-induced inflammation enhances Escherichia coli gut colonization through nitrate respiration. mBio 4:e00430-13. doi:10.1128/mBio.00430-13. PMC3705454 23820397

[104] Wiggers H, Crusca E, Silva É, Cheleski J, Torres N, Navarro M. 2017. Identification of anti-inflammatory and anti-hypertensive drugs as inhibitors of bacterial diguanylate cyclases. J Braz Chem Soc 29:297–309. doi:10.21577/0103-5053.20170141.

[105] Díaz-Cruz M, Mendieta J, Monjonell A, Tauler R, Esteban M. 1998. Study of the zinc-binding properties of glutathione by differential pulse polarography and multivariate curve resolution. J Inorg Biochem 70:91–98. doi:10.1016/S0162-0134(98)10003-X.

[106] Mulder N, Apweiler R. 2007. InterPro and InterProScan, p 59–70. *In* Bergman NH (ed), Comparative genomics. Humana Press, New York, NY. doi:10.1007/978-1-59745-515-2_5.

[107] Edgar RC. 2004. MUSCLE: a multiple sequence alignment method with reduced time and space complexity. BMC Bioinformatics 5:113. doi:10.1186/1471-2105-5-113. 15318951 PMC517706

[108] Price MN, Dehal PS, Arkin AP. 2009. FastTree: computing large minimum evolution trees with profiles instead of a distance matrix. Mol Biol Evol 26:1641–1650. doi:10.1093/molbev/msp077. 19377059 PMC2693737

[109] Letunic I. 2015. phyloT: a phylogenetic tree generator. https://phylot.biobyte.de. Accessed 2018.

[110] Humphrey W, Dalke A, Schulten K. 1996. VMD: Visual molecular dynamics. J Mol Graphics 14:33–38. doi:10.1016/0263-7855(96)00018-5. 8744570

[111] Heppner DE, Dustin CM, Liao C, Hristova M, Veith C, Little AC, Ahlers BA, White SL, Deng B, Lam YW, Li J, van der Vliet A. 2018. Direct cysteine sulfenylation drives activation of the Src kinase. Nat Commun 9:4522. doi:10.1038/s41467-018-06790-1. 30375386 PMC6207713

[112] Phillips JC, Braun R, Wang W, Gumbart J, Tajkhorshid E, Villa E, Chipot C, Skeel RD, Kalé L, Schulten K. 2005. Scalable molecular dynamics with NAMD. J Comput Chem 26:1781–1802. doi:10.1002/jcc.20289. 16222654 PMC2486339

[113] Huang J, MacKerell AD. 2013. CHARMM36 all-atom additive protein force field: validation based on comparison to NMR data. J Comput Chem 34:2135–2145. doi:10.1002/jcc.23354. 23832629 PMC3800559

[114] Frisch MJ, Trucks GW, Schlegel HB, Scuseria GE, Robb MA, Cheeseman JR, Scalmani G, Barone V, Petersson GA, Nakatsuji H, Li X, Caricato M, Marenich AV, Bloino J, Janesko BG, Gomperts R, Mennucci B, Hratchian HP, Ortiz JV, Izmaylov AF, Sonnenberg JL, Williams-Young D, Ding F, Lipparini F, Egidi F, Goings J, Peng B, Petrone A, Henderson T, Ranasinghe D, Zakrzewski VG, Gao J, Rega N, Zheng G, Liang W, Hada M, Ehara M, Toyota K, Fukuda R, Hasegawa J, Ishida M, Nakajima T, Honda Y, Kitao O, Nakai H, Vreven T, Throssell K, Montgomery JA, Jr, Peralta JE, Ogliaro F, et al. 2010. Gaussian 09, revision B.01. Gaussian, Inc, Wallingford, CT.

[115] Becke AD. 1993. Density‐functional thermochemistry. III. The role of exact exchange. J Chem Phys 98:5648–5652. doi:10.1063/1.464913.

[116] Dolg M, Wedig U, Stoll H, Preuss H. 1987. Energy‐adjusted ab initio pseudopotentials for the first row transition elements. J Chem Phys 86:866–872. doi:10.1063/1.452288.

[117] Rassolov VA, Pople JA, Ratner MA, Windus TL. 1998. 6–31G* basis set for atoms K through Zn. J Chem Phys 109:1223–1229. doi:10.1063/1.476673.

[118] Hariharan PC, Pople JA. 1973. The influence of polarization functions on molecular orbital hydrogenation energies. Theor Chim Acta 28:213–222. doi:10.1007/BF00533485.

[119] Ditchfield R, Hehre WJ, Pople JA. 1971. Self‐consistent molecular‐orbital methods. IX. An extended Gaussian‐type basis for molecular‐orbital studies of organic molecules. J Chem Phys 54:724–728. doi:10.1063/1.1674902.

[120] Zhao Y, Truhlar DG. 2008. The M06 suite of density functionals for main group thermochemistry, thermochemical kinetics, noncovalent interactions, excited states, and transition elements: two new functionals and systematic testing of four M06-class functionals and 12 other functionals. Theor Chem Account 120:215–241. doi:10.1007/s00214-007-0310-x.

[121] Schindelin J, Arganda-Carreras I, Frise E, Kaynig V, Longair M, Pietzsch T, Preibisch S, Rueden C, Saalfeld S, Schmid B, Tinevez JY, White DJ, Hartenstein V, Eliceiri K, Tomancak P, Cardona A. 2012. Fiji: an open-source platform for biological-image analysis. Nat Methods 9:676–682. doi:10.1038/nmeth.2019. 22743772 PMC3855844

[122] Heinig M, Frishman D. 2004. STRIDE: a web server for secondary structure assignment from known atomic coordinates of proteins. Nucleic Acids Res 32:W500–W502. doi:10.1093/nar/gkh429. 15215436 PMC441567

