# Supplementary figures and images for: A Bacterial Inflammation Sensor Regulates c-di-GMP Signaling, Adhesion, and Biofilm Formation
Source: mBio. 2021 Jun 22;12(3):e00173-21. doi: 10.1128/mBio.00173-21 (PMC8262984; doi:10.1128/mBio.00173-21)

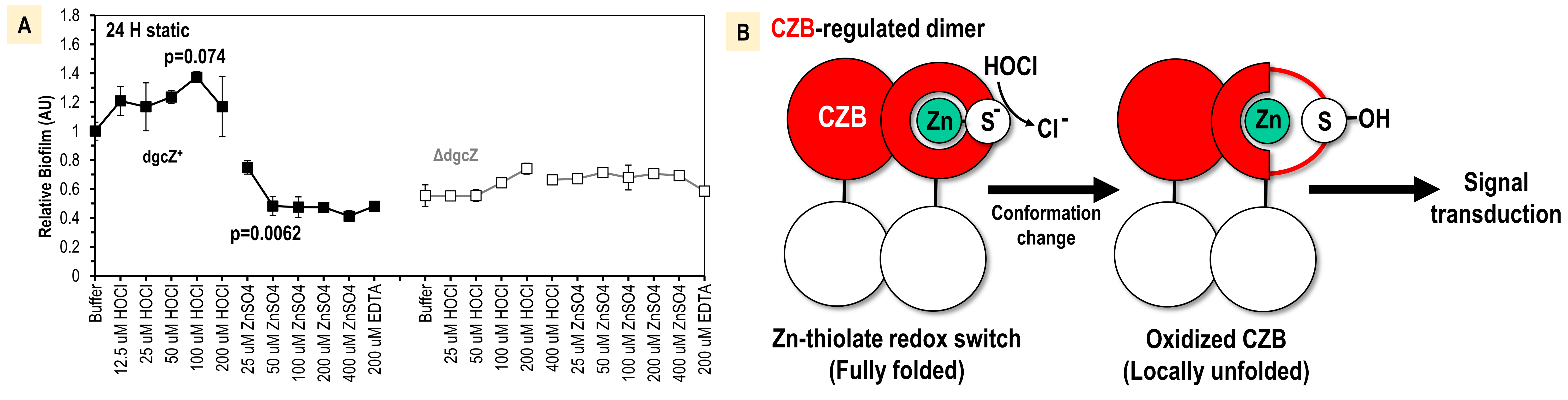

Supplement: FIG S1 [file mbio.00173-21-sf001.tif]

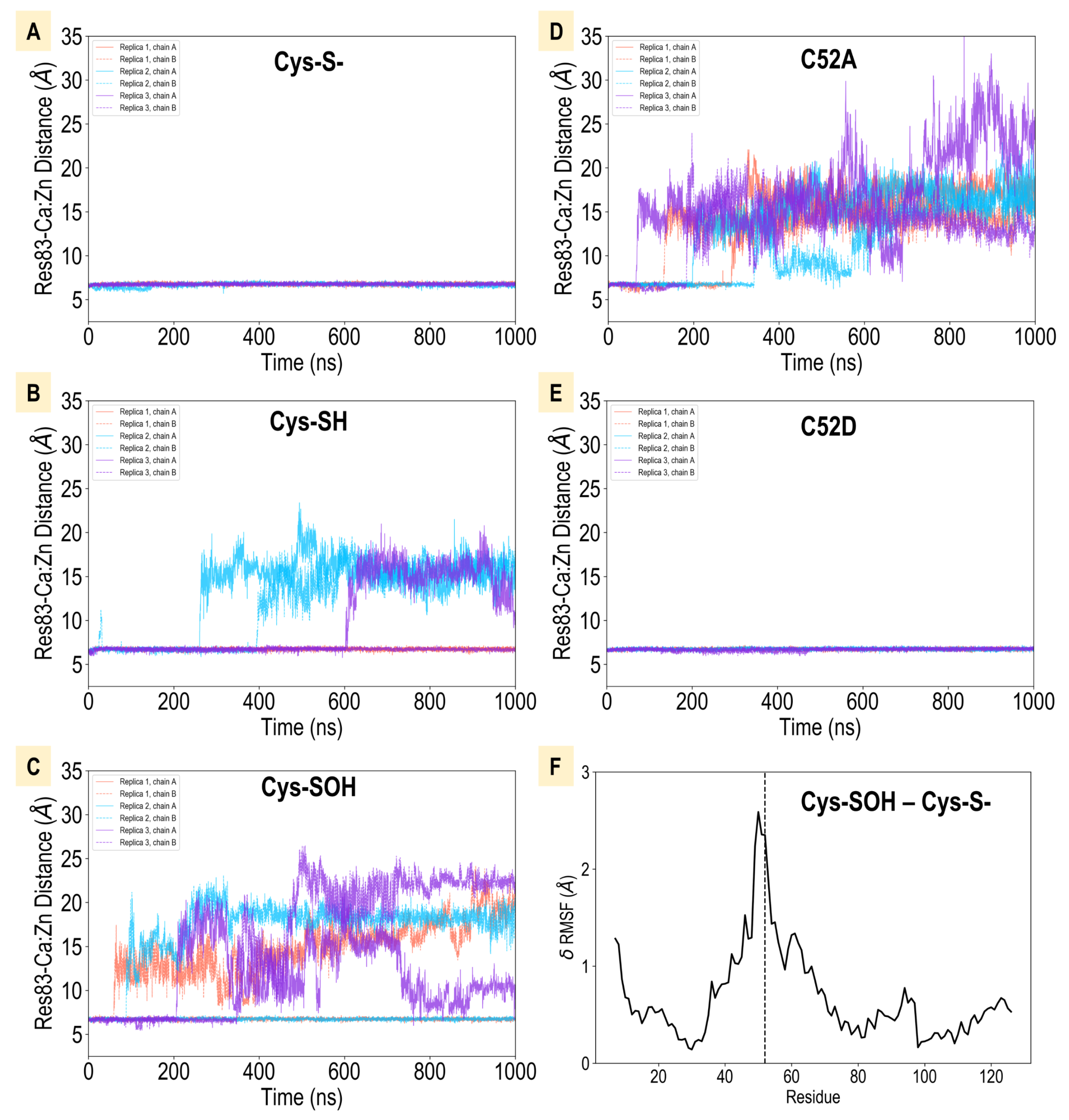

Supplement: FIG S2 [file mbio.00173-21-sf002.tif]

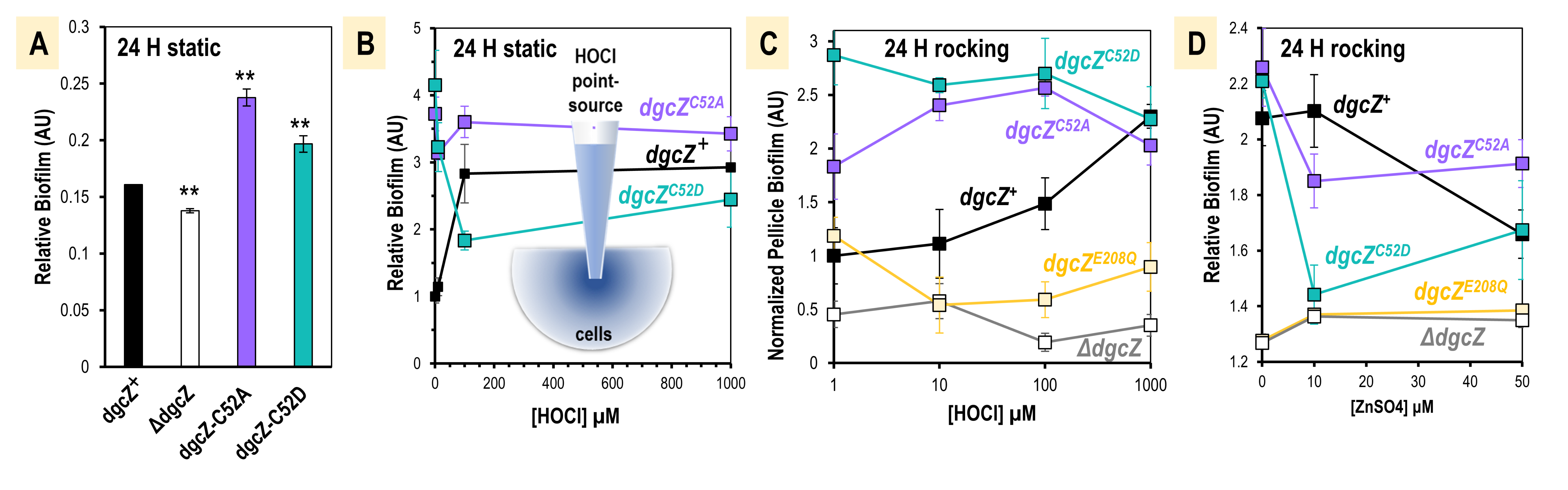

Supplement: FIG S3 [file mbio.00173-21-sf003.tif]

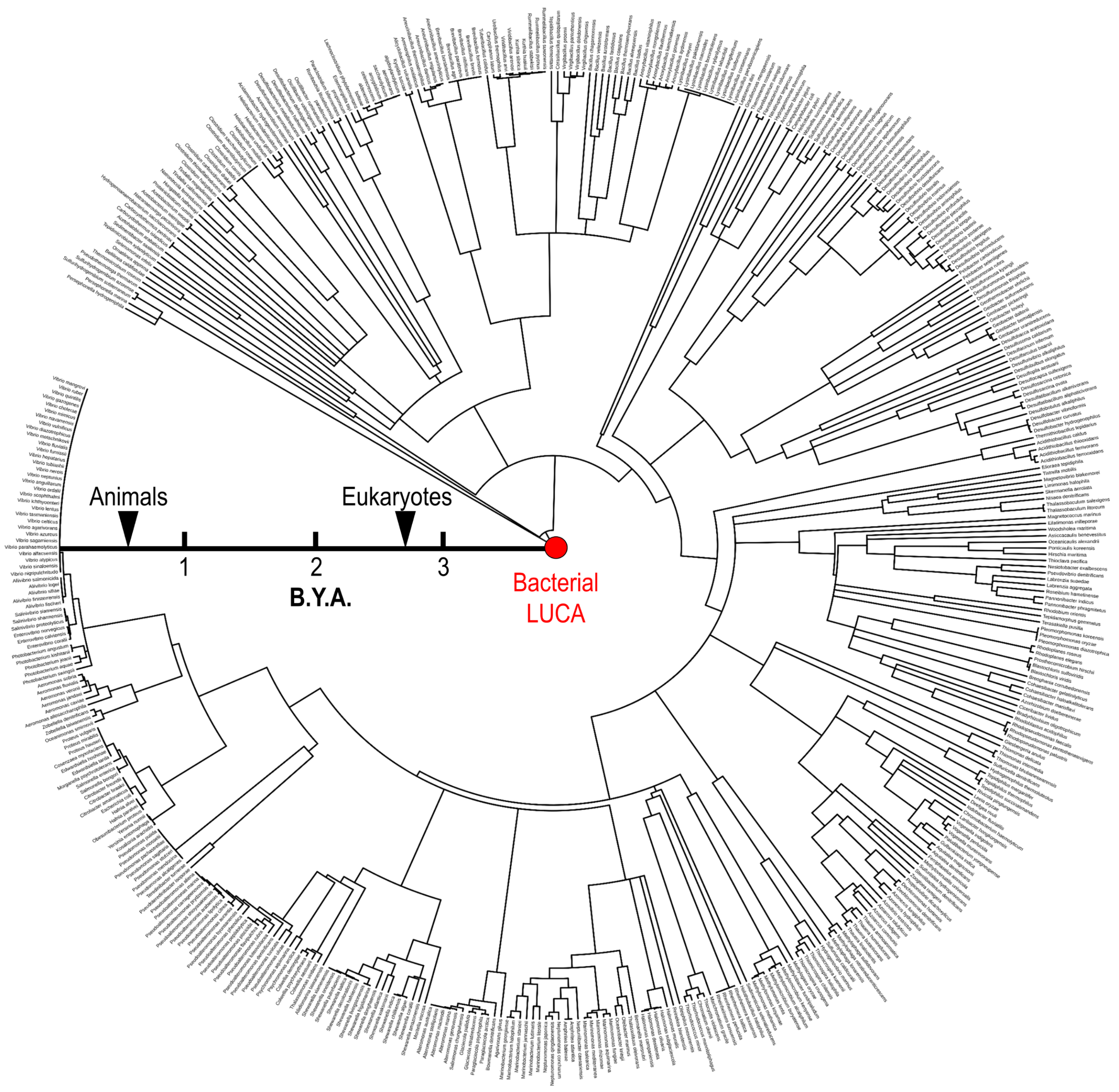

Supplement: FIG S4 [file mbio.00173-21-sf004.tif]
